# Identifying potential exposure reduction priorities using regional rankings based on emissions of known and suspected carcinogens to outdoor air in Canada

**DOI:** 10.1186/s12940-015-0055-2

**Published:** 2015-08-22

**Authors:** Eleanor M. Setton, Basil Veerman, Anders Erickson, Steeve Deschenes, Roz Cheasley, Karla Poplawski, Paul A. Demers, C. Peter Keller

**Affiliations:** Spatial Sciences Research Lab, University of Victoria – Geography, PO Box 3060 STN CSC, Victoria, BC V8W 3R4 Canada; Occupational Cancer Research Centre, Cancer Care Ontario, 525 University Avenue 3rd Floor, Toronto, ON M5G 2L3 Canada

**Keywords:** Canada, Pollution, Toxicity, Health, Provinces, Industry, Transportation, Residential heating

## Abstract

**Background:**

Emissions inventories aid in understanding the sources of hazardous air pollutants and how these vary regionally, supporting targeted reduction actions. Integrating information on the relative toxicity of emitted pollutants with respect to cancer in humans helps to further refine reduction actions or recommendations, but few national programs exist in North America that use emissions estimates in this way. The CAREX Canada Emissions Mapping Project provides key regional indicators of emissions (total annual and total annual toxic equivalent, circa 2011) of 21 selected known and suspected carcinogens.

**Methods:**

The indicators were calculated from industrial emissions reported to the National Pollutant Release Inventory (NPRI) and estimates of emissions from transportation (airports, trains, and car and truck traffic) and residential heating (oil, gas and wood), in conjunction with human toxicity potential factors. We also include substance-specific annual emissions in toxic equivalent kilograms and annual emissions in kilograms, to allow for ranking substances within any region.

**Results:**

For provinces and territories in Canada, the indicators suggest the top five substances contributing to the total toxic equivalent emissions in any region could be prioritized for further investigation. Residents of Quebec and New Brunswick may be more at risk of exposure to industrial emissions than those in other regions, suggesting that a more detailed study of exposure to industrial emissions in these provinces is warranted. Residential wood smoke may be an important emission to control, particularly in the north and eastern regions of Canada. Residential oil and gas heating, along with rail emissions contribute little to regional emissions and therefore may not be an immediate regional priority.

**Conclusions:**

The developed indicators support the identification of pollutants and sources for additional investigation when planning exposure reduction actions among Canadian provinces and territories, but have important limitations similar to other emissions inventory-based tools. Additional research is required to evaluate how the Emissions Mapping Project is used by different groups and organizations with respect to informing actions aimed at reducing Canadians’ potential exposure to harmful air pollutants.

**Electronic supplementary material:**

The online version of this article (doi:10.1186/s12940-015-0055-2) contains supplementary material, which is available to authorized users.

## Background

Exposure to hazardous pollutants in outdoor air is ubiquitous, affecting large populations and impacting health [1–4]. In conjunction with measuring pollutant levels in outdoor air, understanding the sources of pollutants, their relative and cumulative contribution to potential health impacts, and how these vary regionally is key to developing targeted emissions reduction strategies [5].

Emissions inventories are commonly used to estimate the relative contributions of various sources of pollutants in one or more geographic areas. Emitters are typically spatially referenced and categorized as point sources (e.g., industrial and commercial operations at specific locations), line sources (e.g., transportation), and area sources (e.g., agriculture). In Canada, the federal government maintains the National Pollutant Release Inventory (NPRI) and requires industrial and commercial point sources above a certain size to report annual releases and transfers to air, land and water [6]. National emissions inventories that include point, line and area sources are prepared by the federal government [7], and more detailed regional or local emissions inventories are sometimes prepared by provincial and municipal governments [8, 9].

While emissions inventories provide readily available information on the relative contribution of many different sources to total emissions for individual pollutants, there are limitations in using them for directly prioritizing population exposure reduction activities. Firstly, the emission levels may not be based on actual monitoring data at each site, but on industrial production levels or fuel usage in combination with emission factors, which provides only estimates of emissions amounts [10]. Emission factors can be difficult to establish (or missing) for many pollutants, and can be out of date if technologies change [11]. Secondly, the amount of a substance emitted is a coarse indicator of population exposure. Many factors influence the actual concentration of a pollutant in outdoor air, including wind speed and direction at the time of emission, and how quickly the pollutant degrades or settles. These dispersion factors affect the spatial pattern of pollution levels and in turn, how many people are actually exposed. Thirdly, the toxicity of each pollutant is an important consideration when prioritizing exposure reduction activities. A small emission of a highly toxic pollutant may be a higher priority than a large emission of a relatively benign pollutant.

In North America, only one national initiative has used a detailed emissions inventory in combination with both air quality modelling and toxicity information to develop indicators for ranking pollutant emissions based on potential health impacts. The United States Environmental Protection Agency (US EPA) conducts ongoing evaluations of a wide range of air pollutants under the National-Scale Air Toxics Assessment (NATA) program [12]. Four national assessments have been completed to date (1996, 1999, 2002 and 2005), each providing estimates of ambient concentrations of toxic air pollutants for most census tracts in the US and the associated cancer and non-cancer chronic risks (when possible and applicable) for their residents. In 2005, census tract-level ambient concentration estimates for 177 air pollutants were produced by modelling the dispersion of emissions from a variety of sources, including large industries as reported to the Toxic Release Inventory (TRI), small point sources such as gas stations and dry cleaners, on- and off-road vehicle traffic, as well as marine vessels and trains. Given estimated ambient concentrations, long-term health risks (cancer and non-cancer) were estimated by calculating exposure (intake) and applying cancer risk factors or non-cancer hazard quotients to the resulting intake level. This process enables the ranking of air pollutants based on lifetime excess cancer risk or non-cancer hazard indices in each census tract.

Other national initiatives exist but are not as comprehensive as NATA. Scorecard [13], a non-governmental initiative in the US, presents hazard indicators based on the 1996 NATA estimates of ambient concentrations in conjunction with their own selection of cancer and non-cancer potency factors and, in some cases, more current population data (year 2000). Unlike the NATA indicators, the Scorecard indicators do not calculate exposure using standard breathing rates and time spent outdoors, but instead treat the estimated ambient concentration as equivalent to individual exposure. Taking Stock Online [14], developed by the Commission for Environmental Cooperation (CEC), provides a synthesis of emissions reported to the Canadian NPRI, the US TRI, and Mexico's Registro de Emisiones y Transferencia de Contaminates (RETC), but does not include emissions from other non-reporting sources (i.e., transportation). The same cancer and non-cancer potency factors used by Scorecard are applied to emission amounts to produce toxic equivalent emissions which are presented as risk scores. In Canada, Pollution Watch uses only NPRI data and so does not include emissions from other potential sources. Pollutants are categorized according to potential health effects (carcinogen, endocrine disruptor, respiratory toxicant, or reproductive/development toxicant). Total emissions, not toxic equivalent emissions, are used as indicators for ranking.

Spatial querying and reporting using online maps is a feature common to NATA, Scorecard, Taking Stock Online and Pollution Watch. NATA produces results for every county in the US and Google Earth files showing indicators for cumulative cancer and non-cancer risk due to inhalation of air toxics can be downloaded state by state [15]. In Google Earth, each county has a pop-up window providing details about the pollutants contributing to the health risk and the major sources. Scorecard provides a clickable map interface on their website to access state-specific reports, and allows zipcodes to be entered to see community-based reports [16]. Taking Stock Online lets users select reporting regions (national and state/province), air pollutants by name or type (e.g., developmental/reproductive toxins or known/ suspected carcinogens) and display the toxic equivalent emissions. In addition, a Google Earth file can be downloaded showing the location of each emitting facility in North America [17]. Pollution Watch includes an option on their website to create a report of emitters by substance type or specific name, or for a residential address and user-defined buffer distance [18].

Canadian information on air pollutants is provided by Pollution Watch and Taking Stock Online, although both initiatives are based solely on emissions reported to the NPRI, omitting potentially large sources of pollutants. For example, in 2011, Environment Canada estimated emissions of fine particulates in Canada to be on the order of 1.18 million tonnes [7]. Of that, 73 % was attributed to dust from agriculture, construction, and transportation; 9 % to residential wood burning; 6 % to forest fires; and only 6 % to industrial activities (of which the NPRI captures only those above the reporting thresholds). Similarly, of the 2 million tonnes of total volatile organic compounds estimated to be emitted by human sources in 2011, 691,000 tonnes were attributed to industrial activities, 470,228 to mobile sources, and 148,425 to residential wood burning. Developing an accurate, spatially detailed emissions inventory of the kind required to support air quality modelling comparable to NATA is a substantial undertaking beyond the scope of many organizations. The goal of understanding which human sources and associated pollutants contribute most to potential health impacts, however, suggests that it would be useful to include at least the most significant sources in addition to industrial activities.

The objectives of this paper are twofold. First, we describe the development and implementation of the CAREX Emissions Mapping Project (EMP) in Canada, a Google Earth-based data set that includes indicators based on emissions of 21 known and suspected carcinogens to air, as reported to the NPRI and from our own estimates of emissions from transportation and residential heating, circa 2011 [19]. The EMP thus represents an intermediate level between those initiatives using only reported industrial emissions, and those requiring highly detailed spatial data on emissions and expertise in dispersion modelling. Secondly, we illustrate the use of the indicators for identifying potential exposure reduction priorities among provinces and territories in Canada. In general, the indicators should be considered as analogous to screening-level risk or impact assessments. These relatively simple indicators serve only as a first pass, helping to highlight substances or sources that may be of concern, and therefore warrant additional attention through more detailed analyses.

## Methods

Our approach was informed by four key goals: 1) to be national in scope; 2) to support prioritization of potential exposure reduction activities both among substances and geographic regions; 3) to support surveillance of trends; and 4) to use only publicly available existing data.

We use total toxic equivalent emissions (Total TEQ) as the overall indicator for the EMP, which is the sum of annual emissions to air, in toxic equivalent kilograms of benzene, for 17 of the 21 selected known and suspected carcinogens. The indicator is meant to illustrate differences in Canadians’ potential exposure to these substances in outdoor air for the year 2011. Total TEQ was calculated from data reported to the National Pollutant Release Inventory (NPRI) and our own estimates of emissions from transportation (airports, trains, and car and truck traffic) and residential heating (oil and gas, wood), in conjunction with human toxicity potential factors developed by EG Hertwich et al. [20]. We also include substance-specific annual emissions in toxic equivalent kilograms (TEQ), and annual emission in kilograms (TE), to allow for ranking substances within any region. TE was calculated for four additional substances of interest that were not included in Total TEQ or TEQ due to the lack of a toxicity factor.

The indicators were calculated for Canada as a whole (*n* = 1), each province or territory (*n* = 13), ecozones (*n* = 15) health regions (*n* = 133), watersheds (*n* = 594), and within 25 km of major cities (*n* = 159). We chose to provide the indicators for different region types for two reasons: first, the larger regions provide the indicators for areas of interest to policy makers and regulators (provinces/territories, health regions, watersheds and ecoregions), and; secondly, the city-level indicators (within 25 km of city centres) provide a better level of spatial detail in terms of population locations. For example, a user could look at the indicators for health regions, and overlay the indicators for major cities to get a better understanding of the potential population exposure.

In addition to the indicators, we provide geo-referenced files of the major industrial emitters that can be overlaid with the region files, again to provide a visualization of the relative size of emissions reported and proximity to population centres. Although not included in the calculation of the indicators, geo-referenced files of federally-listed contaminated sites [21] and mine tailings and waste rock sites [22] are also made available.

All data were acquired from publicly available websites and processing steps for calculating the indicators are discussed here briefly. More detailed information is provided in the Additional file 1.

### Industrial emissions estimates

In Canada, all companies and organizations that emit certain substances to air, water or land and meet certain threshold requirements must report annual emissions to the federal NPRI. There are currently 362 substances on the NPRI list [23]. For most substances, reporting requirements are based on the number of employees at the facility; the quantity of the substance(s) manufactured, processed, used or released; and the type of activities performed at the facility. Although comprehensive, the NPRI does not include emissions from any activities that do not meet the reporting requirements. These typically include small operations and facilities across many sectors, such as “forest product manufacturing facilities, foundries, rubber and plastics manufacturing plants, transportation equipment manufacturing facilities (except major automobile assembly plants), conventional oil and gas extraction facilities, pits and quarries, certain types of mines, and wastewater facilities” [24]. In some cases, the amount of emissions reported is based on monitoring data. In other cases, emissions are estimated using production levels and emission factors. Guidance in preparing these estimates is provided and emission factors are typically based on or are similar to those published by the United Stated Environmental Protection Agency or the Australia National Pollutant Inventory guides [25]. Total annual emissions for 21 known or suspected carcinogens (Table 1) were extracted by facility, along with geographic coordinates, from the NPRI Excel file for 2011. All amounts were converted to kilograms to produce TE, which was then multiplied by the appropriate toxic equivalent factor to produce TEQ.

**Table 1:** Summary of available data for emissions and toxic equivalent factors by substance and source

Substance	Carcinogen category^a^	NRPI	Vehicles	Trains	Airplanes	Residential-oil	Residential-gas	Residential-wood	Toxic Equivalent factor
Y = Data available *N* = data not available NO EF = No emission factors found NO TEQ = no toxic equivalent factor									
1,3,-butadiene	1	Y	Y	Y	Y	NO EF	NO EF	Y	0.54
Arsenic	1	Y	NO EF	Y	Y	NO EF	Y	Y	2600
Benzene	1	Y	Y	Y	Y	NO EF	Y	Y	1
Cadmium	1	Y	Y	Y	Y	Y	Y	Y	28
Fine particulate	1	Y	Y	Y	Y	Y	Y	Y	NO TEQ
Formaldehyde	1	Y	Y	Y	Y	NO EF	Y	Y	0.02
Hexavalent Chromium	1	Y	Y	Y	Y	NO EF	NO EF	Y	NO TEQ
Nickel	1	Y	Y	Y	Y	NO EF	Y	Y	2.8
TCDD	1	Y^b^	NO EF	NO EF	NO EF	Y	NO EF	Y	1,200,000,000
Lead	2A	Y	Y	Y	Y	Y	NO EF	Y	28
Tetrachloroethylene	2A	Y	NO EF	NO EF	--	--	--	--	0.92
Acetaldehyde	2B	Y	Y	Y	Y	--	--	Y	0.017
Benzo[a]anthracene	2B	Y	NO EF	NO EF	NO EF	NO EF	Y	Y	54
Benzo[a]pyrene	2B	Y	NO EF	NO EF	NO EF	Y	Y	Y	6400
Benzo[b]fluoranthene	2B	Y	NO EF	NO EF	NO EF	Y	Y	Y	130
Benzo[k]fluoranthene	2B	Y	NO EF	NO EF	NO EF	Y	Y	Y	NO TEQ
Chlorform	2B	Y	NO EF	NO EF	NO EF	--	--	--	1.6
Chrysene	2B	Y	NO EF	NO EF	NO EF	NO EF	NO EF	Y	5.1
Dichloromethane	2B	Y	NO EF	NO EF	--	--	--	--	0.2
Ethylbenzene	2B	Y	Y	Y	Y	NO EF	NO EF	NO EF	NO TEQ
Indeno[1,2,3-cd]pyrene	2B	Y	NO EF	NO EF	NO EF	Y	Y	Y	280

^a^International Agency for Research on Cancer (IARC) categories: Known carcinogen (1), probable carcinogen (2A) and possible carcinogen (2B)

^b^TCDD is included in the NPRI, but no emissions were reported for 2011

### Transportation and residential heating estimates

These estimates rely on combining activity data (i.e., vehicle kilometres travelled or number of landings and takeoffs at airports) with emission factors (i.e., grams of substance emitted per vehicle kilometre travelled) specific to each substance and sometimes specific to fuel type as well. In some cases, estimates were made for a class of substances (i.e., volatile organic compounds) and then speciation factors were applied (i.e., grams of benzene per kilogram of volatile organic compound). We use Canadian activity data collected by either Statistics Canada or Environment Canada, with the exception of railway activity which was reported by the Railway Association of Canada. Emission factors and speciation factors were not available from a single source for all our substances of interest. We used factors reported in the Environment Canada emissions inventory guidebook [26] when possible, but also used factors from the United States Environmental Protection Agency and the Australian National Pollution Inventory program, both of which are recommended as appropriate sources by Environment Canada for developing emissions estimates [25]. For residential heating, we also undertook a literature review to identify emission factors for as many substances on our list as possible.

#### Airports

Flight type and volume statistics for all major airports (*n* = 98) in Canada for 2011 were acquired from Statistics Canada's annual Aircraft Movement Statistics report [27]. Only airplanes taking off and landing were included in our estimate. Emissions from airplanes flying at altitude and emissions from ground equipment at airports were not included. Based on the type of flight (local or itinerant), general destination (international or domestic) and for domestic flights, percent of light weight planes versus medium weight planes, total hydrocarbons and total suspended particulate emissions in kilograms were estimated using factors from Environment Australia [28], which provided the most comprehensive set of factors for our selected substances. These were further broken down into substance specific totals using speciation factors from the same report. Table 1 indicates which substances' emissions were estimated for airplanes. Airport names, provided in the Statistics Canada data, were matched to a spatial file of airports from DMTI Spatial [29], acquired under the Data Liberation Initiative agreement with Canadian educational institutions.

#### Trains

We used total litres of diesel fuel consumed in Canada in 2011, as reported in the Locomotive Emissions Monitoring Program 2010 report [30], in conjunction with a spatial file providing the geographic location and lengths of railways in operation in 2011 from DMTI Spatial [29]. Fuel consumption was assigned to each rail segment proportionally, and emissions factors from Environment Australia were used to calculate total emissions for 11 of the 22 substances (Table 1). We were not able to find any provincial volume data that would aid in refining this estimate. All kilometers of railway are treated as having the same volume of rail traffic, which may under- or over-estimate emissions for any one railway segment.

#### Car and truck traffic

Provincial and territorial data on vehicle kilometers travelled (VKT) by cars (light duty vehicles) and trucks (heavy duty vehicles) on local roads and highways, along with fuel types (gas, diesel, other) were available for 2009 from Statistics Canada [31]. Emission factors from Environment Australia and from literature review were used with VKT's for each category to calculate emissions for 10 of 22 substances (Table 1) for each province and territory [32]. In order to allocate the emissions more closely to the population centres generating vehicle traffic, we then used a two-step process. First, we used the 2011 Statistics Canada digital block population file [33] and the 2011 Statistics Canada digital population ecumene file [34], which identifies all areas in Canada with a minimum population density of 0.4 persons per square kilometer, to allocate the total provincial emissions to areas within the ecumene and outside the ecumene in proportion to the total population residing in each. Secondly, using a digital file of Canadian roadways from DMTI Spatial [29], we calculated the emissions per kilometer of roadways to get a specific emission factor for each area (within the ecumene and outside the ecumene in each province and territory). We were unable to find enough actual traffic volume data, which would increase the spatial accuracy of the emissions estimates. Our method may overestimate emissions for areas within the ecumene that are at the lower limit of population density, and underestimate the emissions for areas within the ecumene that are very densely populated, such as major urban centres. Still, this method is an improvement to assigning all provincial road segments the same emission factors.

#### Residential heating

Provincial and territorial data on consumption of natural gas and fuel oil for residential heating purposes in 2011 were acquired from Statistics Canada [35], and data on consumption of wood for residential heating by appliance type (e.g., fireplaces, inserts, stoves, or boilers) were reported by Environment Canada [26]. Information on the percentage of dwellings using gas, oil or wood for heating in major Census Metropolitan Areas (CMAs) and non-CMAs in Canada was available for 2006 from Statistics Canada [36] and was incorporated to help allocate the consumption levels more accurately with each province and territory. In addition, we used the digital block population file from Statistics Canada [33] with associated dwelling counts to identify the proportion of dwellings in CMAs and non-CMAs. Fuel consumption (gas, oil, and wood) was allocated to each provincial or territory CMA or non-CMA street block using the proportion of dwellings. Emission factors specific to oil and gas consumption, and wood combustion by appliance type were synthesized from a variety of sources [26, 37–45]. We were not able to estimate emissions due to residential heating for all substances (Table 1).

#### Toxic equivalent factors

We used the toxic equivalent factors derived by EG Hertwich et al. [20]. Briefly these factors were derived using a:

“fate and exposure model which determines the distribution of a chemical in a model environment and accounts for a number of exposure routes, including inhalation of gases and particles, ingestion of produce, fish, meat and dermal contact with water and soil.”[20]

Thus, the toxic equivalent factors take potential exposure levels into account based on the substances’ physical properties. Substances with higher potential for human exposure due to persistence in exposure pathways have a higher toxic equivalent factor. All toxic equivalent factors are expressed relative to benzene, which allows for subsequent summing of toxic equivalent emissions. These were available only for 17 of the 21 substances of interest (Table 1), so the indicators Total TEQ and TEQ are based only on 17 substances.

#### Emissions estimates comparability

We compared our national results for seven substances included in both our estimates and the more detailed Environment Canada (EC) inventory for 2011 [7] (Table 2). Our estimate of total cadmium emissions is 3.3 times higher than the EC estimate. We attribute this difference to the lack of a cadmium estimate for on-road transportation in the EC inventory. In addition, our estimate of cadmium from residential heating (11,014 kg) is much higher than that reported in the EC inventory (662 kg), although we used the emission factors reported in the Environment Canada Criteria Air Contaminants Emissions Inventory 2006 Guidebook [26]. We are unable to account for this discrepancy and were unable to confirm the published emissions factors with Environment Canada staff. Our estimates for lead, benzo[b]fluoranthene, and fine particulates are lower but within a factor of 2 compared to the EC estimates which we consider to be reasonable agreement. Our estimates are more than a factor of 2 lower than the EC estimates for benzo[k]fluoranthene, benzo[a]pyrene and indeno (1,2,3-cd)pyrene. We attribute this difference to fewer sources being included in our estimates.

**Table 2:** Comparison of emission estimates in Canada

Substance	Environment Canada (EC) 2011(kg)	CAREX 2011 (kg)	Comparison
Cadmium	7,953	26,086	CAREX 3.3 times higher
Lead	178,228	169,495	EC 1.05 times higher
Benzo[b]fluoranthene	32,641	18,351	EC 1.8 times higher
Fine particulates (PM_2.5_)	245,650,000	171,838,236	EC 1.4 times higher
Benzo[k]fluoranthene	11,791	5,410	EC 2.2 times higher
Benzo[a]pyrene	19,543	8,434	EC 2.3 times higher
Indeno(1,2,3-cd)pyrene	14,498	6,254	EC 2.3 times higher

These comparisons illustrate some of the challenges in conducting and comparing emissions inventories. The inclusion of different sources, the use of different source activity data, and the use of different emission factors can all contribute to non-agreement between emissions inventories. Still, internal comparisons between regions are valid when the same source data sets and emission factors are used consistently.

## Results

We present results here for ten provinces and three territories as an example of the indicators and their utility in illustrating regional differences in emissions circa 2011. For each included region, the indicators are: the total of all substances’ annual toxic equivalent emissions to air in kilograms (Total TEQ), the annual toxic equivalent emissions to air in kilograms for each substance (TEQ), and the annual emissions to air in kilograms for each substance (TE). TE is also available for each substance by source: industrial activities, on-road vehicles, trains, airplanes taking off and landing, and residential oil, gas and wood heating.

In terms of Total TEQ, the provinces of Quebec and Ontario were ranked first and second respectively, due to the concentration of Canadian industrial, transportation and residential heating activities in these two provinces (Table 3). Interestingly, while Ontario is home to 38 % of Canada’s population, the higher ranked province of Quebec is home to only 24 % [46]. Similarly, the province of New Brunswick ranks third, but only 2.2 % of Canada's population lives in this province. Since our emission estimates for transportation and residential heating are population weighted, high ranks for provinces with lower populations suggests that industrial sources likely dominate the ranking. In fact, 88 % of New Brunswick's indicator is due to emissions reported to the NPRI (Table 4), the highest of any province or territory. Quebec also has a high proportion of industrial emissions (71 % of indicator total) compared to other sources. Table 4 also shows that while rail, residential gas heating and residential oil heating contribute very little to the overall rankings for any of the provinces and territories, residential heating with wood can be a significant source of harmful pollutants (e.g., the northern territories and the Maritime provinces), relative to other sources.

**Table 3:** Rank and population by province and territory

Rank	Province/Territory	2011 population (thousands) [percent of national total]	Indicator: total TEQ (kg)
1	Quebec	8,007.7 [23.6]	93,966,697
2	Ontario	13,263.5 [38.4]	55,718,108
3	New Brunswick	755.5 [2.2]	24,757,165
4	British Columbia	4,449.1 [13.1]	23,462,727
5	Alberta	3,790.2 [10.9]	10,570,015
6	Newfoundland/Labrador	525.0 [1.5]	4,804,302
7	Manitoba	1,233.7 [3.6]	4,531,379
8	Nova Scotia	944.5 [2.8]	3,786,719
9	Saskatchewan	1,066.3 [3.1]	2,757,480
10	Prince Edward Island	144.0 [0.4]	453,195
11	Northwest Territories	43.5 [0.1]	201,167
12	Yukon	35.4[0.1]	133,327
13	Nunavut	34.2 [0.1]	83,538

**Table 4:** Contribution of sources to rank by province and territory

		Percent of Indicator by emission source						
		Industry	Transportation			Residential heating		
Rank	Region	NPRI	Airports	Rail	Roads	Gas	Oil	Wood
1	Quebec	71	1	<1	11	<1	<1	17
2	Ontario	49	3	<1	31	1	<1	15
3	New Brunswick	88	1	<1	5	<1	<1	6
4	British Columbia	54	7	<1	22	<1	<1	17
5	Alberta	14	10	<1	62	2	<1	12
6	Newfoundland Labrador	56	3	<1	14	<1	1	26
7	Manitoba	41	8	<1	35	1	<1	16
8	Nova Scotia	11	5	<1	37	<1	3	44
9	Saskatchewan	4	10	1	62	2	<1	21
10	Prince Edward Island	1	4	0	40	<1	6	48
11	Northwest Terr.	2	53	<1	19	1	2	22
12	Yukon	0	29	<1	45	1	1	24
13	Nunavut	<1	63	0	6	0	0	31

**Fig. 1:**
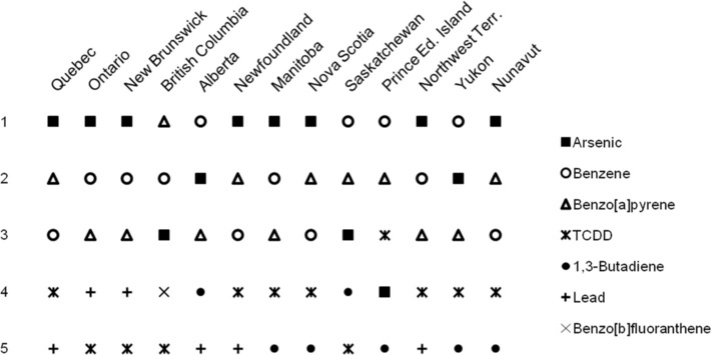
Rank of top 5 substances by province and territory

While we include toxic equivalent emission estimates for 17 substances, in general, between 94 and 99 % of Total TEQ for each province or territory is contributed by only five substances, based on the associated TEQ ranks (Table 5). Arsenic was most frequently the top contributor to Total TEQ, followed by benzene, benzo[a]pyrene, 2, 3, 7, 8-tetrachlorodibenzo -p-dioxin (TCDD), 1,3-butadiene, lead and benzo[b]fluoranthene (Fig. 1). The substance-specific toxic equivalent factor plays an important role - arsenic, benzo[a]pyrene, and especially TCDD have high toxic equivalent factors (see Methods section) which elevate the influence of the relatively small amounts emitted (indicated by TE for each substance). Interestingly, the top five substances vary among provinces and territories. No more than two provinces or territories share the same top five substances in the same order (Quebec and Newfoundland/Labrador; Ontario and New Brunswick; Nova Scotia and Nunavut), and seven have unique lists when order is considered.

**Table 5:** Total TEQ of top 5 substances by province and territory

Rank	Province/Territory	Total TEQ (kg)	TEQ (kg) of top 5 substances	Top 5 TEQ (kg) as percent of Total TEQ (kg)
1	Quebec	93,966,697	90,063,593	96
2	Ontario	55,718,108	53,149,650	95
3	New Brunswick	24,757,165	24,451,448	99
4	British Columbia	23,462,727	22,087,689	94
5	Alberta	10,570,015	10,095,747	96
6	Newfoundland Labrador	4,804,302	4,615,482	96
7	Manitoba	4,531,379	4,276,081	94
8	Nova Scotia	3,786,719	3,604,741	95
9	Saskatchewan	2,757,480	2,617,953	95
10	Prince Edward Island	453,195	429,651	95
11	Northwest Territories	201,167	189,937	94
12	Yukon	133,327	125,988	94
13	Nunavut	83,538	80,111	96

Regional variation in Total TEQ and substance TEQ ranks reflect the differences in sources. For example, Table 6 shows the contribution of each substance to the total of the top five TEQ. Clearly, in New Brunswick, arsenic is responsible for 88 % of the province's relatively high overall ranking, and as per Table 4, industrial sources are the major contributor. Additional exploration is required to better understand sources in the top-ranked province of Quebec, where only 50 % of the ranking is due to arsenic. Table 7 shows the relative contribution of each source to the top five substances in Quebec specifically. In this case, arsenic from industrial sources (as reported to the NPRI) makes up 97 % of the total arsenic TEQ. In contrast, roughly two thirds of the benzo[a]pyrene TEQ is associated with industrial sources and one third with residential wood burning, while 87 % of the benzene TEQ is associated with traffic on roads.

**Table 6:** Percent contribution to TEQ of top five substances

		Percent contribution to top 5 TEQ						
Rank	Province	Arsenic	Benzo[a]pyrene	Benzene	TCDD	1,3-butadiene	Lead	Benzo[b]fluoranthene
1	Quebec	51	33	12	2		2	
2	Ontario	48	14	32	2		3	
3	New Brunswick	88	4	5	1		2	
4	British Columbia	18	54	23	2			3
5	Alberta	24	8	62		4	2	
6	Newfoundland Labrador	60	18	16	5		1	
7	Manitoba	49	11	35	2	2		
8	Nova Scotia	17	30	40	10	3		
9	Saskatchewan	12	17	64	3	4		
10	Prince Edward Island	7	32	44	14	3		
11	Northwest Territories	56	15	21	6		2	
12	Yukon	30	16	46	5	3		
13	Nunavut	63	21	10	5	1

**Table 7:** Top 5 substances by source (percent) - Quebec

	Percent contribution to total substance emitted						
Substance	Airports	NPRI	Rail	Res gas	Res oil	Res wood	Roads
Arsenic	2	97	<1	<1	0	1	0
Benzo[a]pyrene	0	65	0	<1	<1	35	0
Benzene	<1	<1	<1	<1	0	13	87
TCDD	0	0	0	0	6	94	0
Lead	<1	88	<1	0	<1	1	10

It will be important to conduct additional investigations to ascertain the likelihood of significant population exposure to these substances, given that the emissions are attributed to such large regions. It is entirely possible that some of the emissions are not released near population centres of any size. While we have attempted to spatially allocate emissions as accurately as possible (i.e., restricting vehicle emissions to areas within the population ecumene), this is not reflected in the provincial/territorial ranks. For this reason, we have calculated the ranks for major cities (*n* = 159), and also provide supplementary files showing the location of large industrial emitters via the Google Earth platform, as described in the following example.

**Fig. 2:**
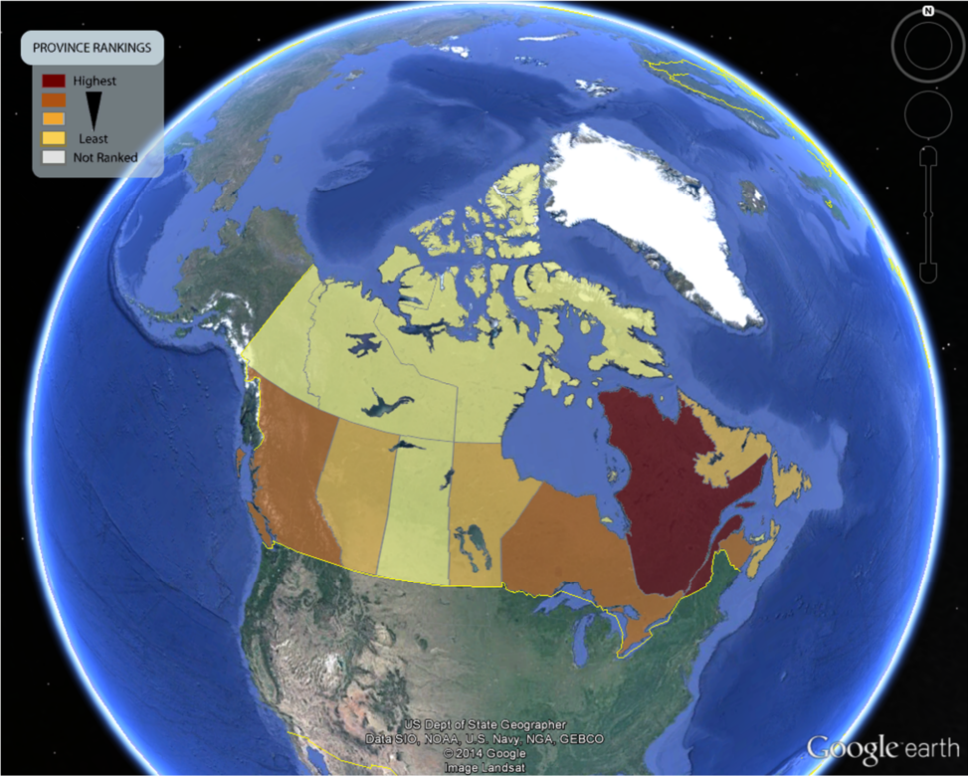
Canadian provinces and territories ranked by total toxic emissions of selected carcinogens to air (2011)

**Fig. 3:**
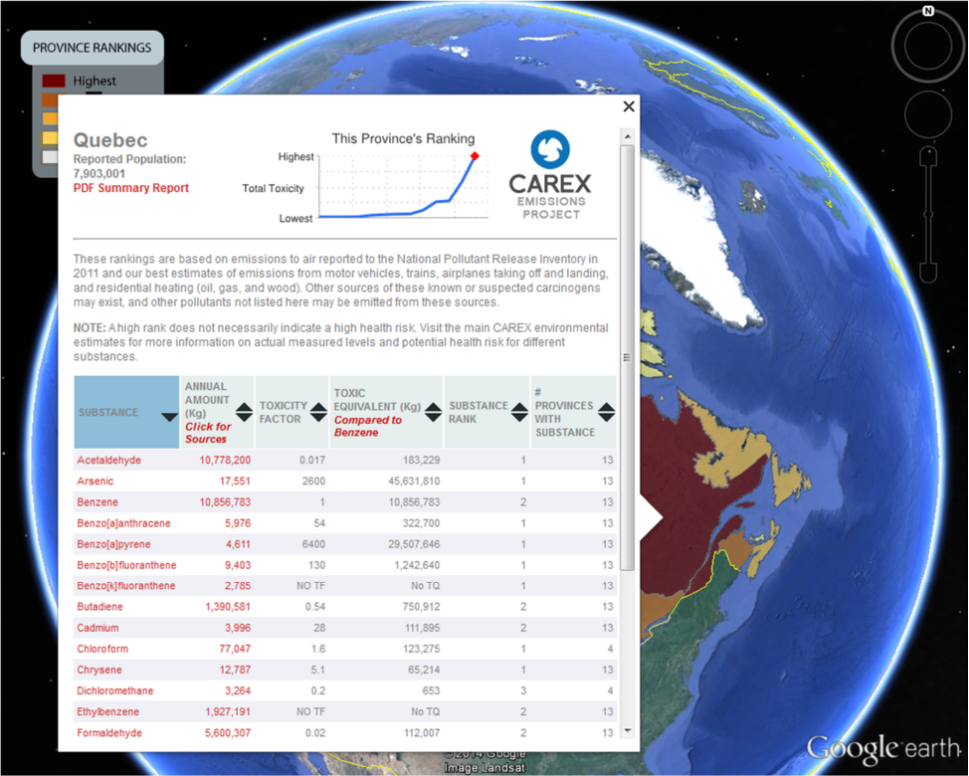
Example of supporting information available for the ranking via Google Earth

The Google Earth platform provides different and more dynamic views of the CAREX EMP results. Opening the provincial ranks file produces an overlay map of each province and territory in Canada, with a simple colour code indicating relative rank order from high to low (Fig. 2). Clicking on any region opens an information box with a simple graph at the top showing the Total TEQ rank compared to all other regions and six columns of data (Fig. 3). The first column lists all included substances (sorted alphabetically). The second column gives TE – the total annual emissions in kilograms. The third column shows the toxic equivalent factor, and the fourth gives TEQ (TE multiplied by the toxic equivalent factor). The fifth and sixth columns gives the numerical rank (i.e., first, second, third, etc.) of the substance TEQ compared to all other regions. For example, in Fig. 3, acetaldehyde has a substance rank of 1 (column 5) out of 13 regions (column 6) in the province of Quebec. This means no other province or region has a higher emission of acetaldehyde. Each of the columns can be sorted by clicking on the column header.

**Fig. 4:**
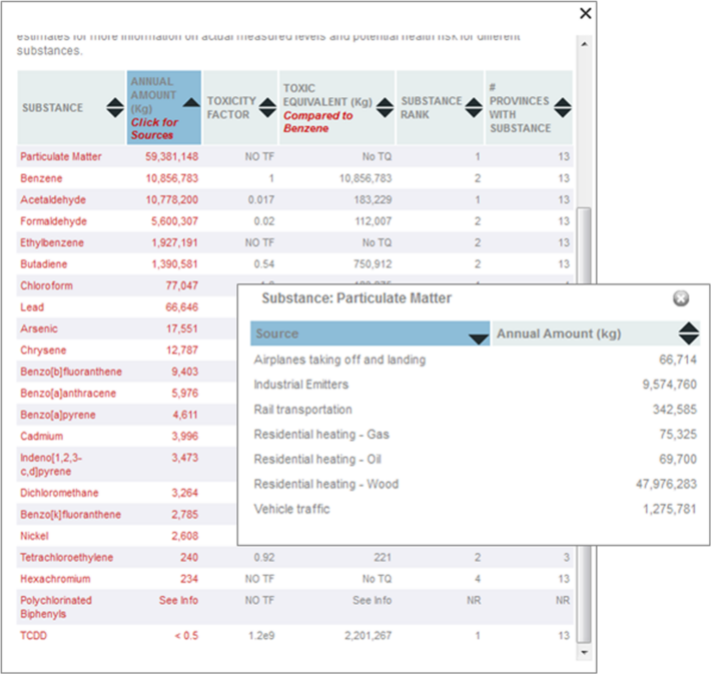
Example of detailed information on sources included in ranking

The information box is the first ‘layer’ of information available to the user. Each of the substance names in the first column are linked to substance profiles on an external website (e.g., http://www.carexcanada.ca/en/benzene/) which include detailed information about the substance. Each of the TE amounts listed in the second column is linked to a pop-up box that gives the TE for each source (Fig. 4). At the top left, users can link to a downloadable report for the region.

**Fig. 5:**
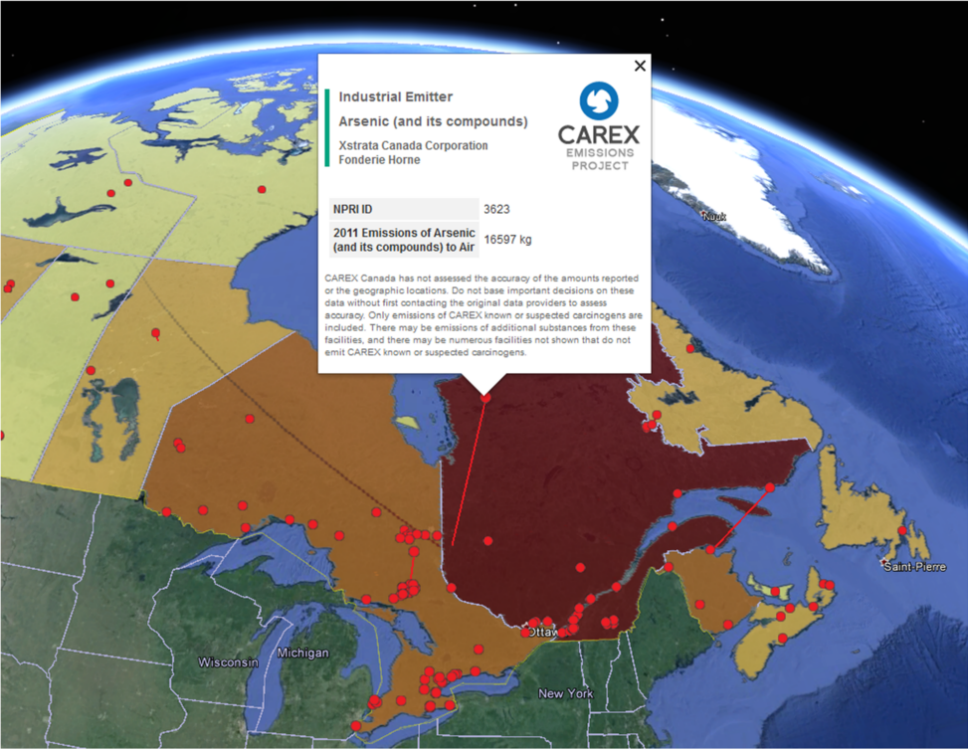
Example of additional Google Earth file providing spatial context for sources within a ranked region

The CAREX EMP includes a range of supporting files in addition to the ranked regions. Feedback from various potential users during development identified the value of having both substance- and source-specific files available to help visualize sources within any particular region. Given that the ranked regions only give the total emissions, these additional files can be used to explore the geographic distribution of sources and their relative emissions within a region. For example, in Quebec, arsenic emissions are the top contributor to Total TEQ. The rank file provides the total amount of arsenic emitted to air – 17,007 kg in 2011 – and the associated file of industrial emitters of arsenic to air shows one major source (16,597 kg in 2011) not in proximity to any major population centres and numerous very small sources (Fig. 5). This may reduce the priority for additional analysis of arsenic as a potential population exposure concern.

Altogether, these views provide easy access to a wide range of information about the known and suspected carcinogens included in the CAREX EMP.

## Discussion

We used publicly available data and Google Earth to develop and implement an information platform (the CAREX EMP) for emissions of selected known and suspected carcinogens in various regions of Canada. This is the first platform of its kind in Canada. Although similar in part to Pollution Watch and Taking Stock which are based on NPRI data, the CAREX EMP includes estimates of emissions from sources not required to report to the NPRI, namely transportation (on-road vehicles, airplanes and trains) and residential heating (oil, gas and wood).

The overall region rank (Total TEQ) and associated substance ranks (TEQ and TE) allow users to make useful relative comparisons among regions and substances. These comparisons can highlight differences in environmental quality, and may have important implications for developing policies or regulations aimed at reducing potential exposures to harmful emissions. The provincial/territorial rankings illustrated in the results give support for a range of priorities, for example: 1) the substances that contribute most to the Total TEQ in any region could be prioritized for further investigation (i.e., the top five substances based on their individual TEQs); 2) residents of Quebec and New Brunswick may be more at risk of exposure to industrial emissions than those in other regions, suggesting that a more detailed study of exposure to industrial emissions in these provinces is warranted (and can be accomplished initially by using additional Google files from the EMP, such as substance-specific emission files; 3) given the relative lack of large industrial emitters in the Maritimes and the north, residential woodsmoke may be the most important emission source to control; and 4) residential oil and gas heating, along with rail emissions contribute little to regional emissions and therefore may NOT be an immediate regional priority.

Perhaps the most critical limitation of our approach is the lack of emission and toxic equivalent factors for some substances of interest (which are therefore omitted from the ranking), as well as the accuracy of those factors that are available. This may have a large impact, as the inclusion of new substances or changes in existing factors could affect the overall indicator and the individual substance rankings in unknown ways. Canada-specific emission factors were not readily available for many substances and sources of interest, and it is difficult to assess how different these might be from the ones used for the EMP. These limitations are not exclusive to the EMP, but would also affect any other summary of emissions and their toxic equivalents, such as emission inventories produced by national or regional governments, or projects like Taking Stock, Pollution Watch or Scorecard.

Other limitations exist. We have only incorporated major sources of emissions. There may be other sources, in which case our estimates would be lower than actual emissions. For example, we did not include emissions from marine transportation, although there are large ports associated with major cities on both coasts and along the St. Lawrence Seaway. According to the EC emissions inventory for 2011 [7], this source contributes less than 1 % of the annual emissions of fine particulates, lead, volatile organic compounds as a group, and polycyclic aromatic hydrocarbons as a group. However, approximately 3.4 and 28 % of annual emissions of cadmium and dioxins/furans respectively are associated with marine transportation. Given the extremely high toxicity factor for the dioxin TCDD, the presence of a port could affect regional rankings. Open burning is also identified as a significant source of dioxins/furans (24 % of the annual total) in the EC emissions inventory. These sources could be added in future updates of the EMP. The geographic boundaries are somewhat arbitrary, and summing total emissions to air within a region does not account for the movement of air across boundaries from nearby sources, or long range transport from distant sources. The indicators do not reflect actual concentrations in ambient air, or individual exposure. At best, the indicator identifies potential for exposure. In contrast, the US EPA NATA program is based on a highly detailed emissions inventory for each county in the US. This inventory is then used as input to an atmospheric dispersion model, which outputs predicted ambient concentrations for each census tract. These concentrations can be combined with population characteristics to estimate the range of exposures likely to be experienced. We opted for relative simplicity in our approach to the EMP, given the scope of funding and the complexities of undertaking national dispersion modelling.

Over time, it is possible that the indicators will change, and it is necessary to have future datasets comparable to those used for 2011 to establish trends. For this reason, we have limited our use of datasets to those that are publicly available from government sources, with a reasonable expectation that they will continue to exist in the future.

Limitations notwithstanding, the benefits of using this approach are twofold: 1) it provides at least an initial indication of which pollutants may be of most concern due to human health impacts, rather than relying solely on total amount emitted; 2) it does not demand the data and expertise required to undertake the dispersion modelling for predicting actual ambient concentrations, but does provide some guidance as to where and on what pollutants this effort might be best focused.

We found no published literature evaluating these types of national tools in terms of their utility in increasing awareness of potentially harmful pollutants, or supporting the prioritization of exposure reduction activities. While today’s technologies make these kinds of information platforms relatively easy to create, more research is required to establish their effectiveness. A number of the authors are currently conducting such research, focusing specifically on evaluating training effectiveness and tool use in selected First Nations organizations in Canada.

## Conclusion

Indicators showing the total emissions and total toxic equivalent emissions can be relatively easy to implement, and may support increasing awareness and evidence for exposure reduction actions with respect to known and suspected carcinogens emitted to air. For example, our results suggest that wood smoke from residential heating may be an important source of cancer-related pollutants, indicating a shift away from focusing on industrial activities may be warranted in some regions of Canada. Importantly, the impact of missing or out-of-date emission factors and toxic equivalent factors is not easily quantifiable, and is a key limitation of this approach. Further research is required to evaluate how users employ the information provided, and what kinds of impacts the EMP has on future activities aimed at reducing potential exposures of the Canadian public.

## Additional file

Additional file 1:
**EMP methods report 2011 final.** (PDF 1205 kb)

## Abbreviations

CEC: Commission for Environmental Cooperation
EMP: Emissions Mapping Project
kg: kilograms
NATA: National Air Toxics Assessment
NPRI: National Pollutant Release Inventory
RETC: Registro de Emisiones y Transferencia de Contaminates
TCDD: 2,3,7,8-tetrachlorodibenzo-p-dioxin
TE: Total emissions
TEQ: Toxic equivalent emissions
TRI: Toxic Release Inventory
US EPA: United States Environmental Protection Agency

## References

[1] Brender JD, Maantay JA, Chakraborty J (2011). Residential proximity to environmental hazards and adverse health outcomes. Am J Public Health.

[2] Henschel S, Atkinson R, Zeka A, Le Tertre A, Analitis A, Katsouyanni K (2012). Air pollution interventions and their impact on public health. Int J Public health.

[3] Kampa M, Castanas E (2008). Human health effects of air pollution. Environ Pollut.

[4] O'Neill MS, Breton CV, Devlin RB, Utell MJ (2012). Air pollution and health: emerging information on susceptible populations. Air Quality, Atmosphere Health.

[5] Ayala A, Brauer M, Mauderly JL, Samet JM (2012). Air pollutants and sources associated with health effects. Air Quality, Atmosphere Health.

[6] Environment Canada. National Pollutant Release Inventory. URL: http://www.ec.gc.ca/inrp-npri/. Access Date: 2015 Jan. 28.

[7] Environment Canada. 2011 Air Pollutant Emission Summaries and Historical Emission Trends. URL: https://www.ec.gc.ca/inrp-npri/default.asp?lang=En&n=D999B315-1. Access Date: 2015 Jan. 28.

[8] British Columbia Ministry of Environment. BC Air Quality Emissions Inventories. URL: http://www.bcairquality.ca/assessment/emissions-inventories.html. Access Date: 2015 July 24.

[9] Metro Vancouver. Emission Inventories and Forecasts. URL: http://www.metrovancouver.org/services/air-quality/emissions-monitoring/emissions/emission-inventories/Pages/default.aspx. Access Date: 2015 July 24.

[10] Environment Canada. National Pollutant Release Inventory Uncertainty Analysis of Emission Estimates for Selected Sectors. URL: http://www.ec.gc.ca/inrp-npri/default.asp?lang=En&n=DBB696D2-1. Access Date: 2015 Jan. 5.

[11] Miller CA, Hidy G, Hales J, Kolb CE, Werner AS, Haneke B (2006). Air emission inventories in north america: a critical assessment. J Air Waste Manage Assoc.

[12] United States Environmental Protection Agency. National Air Toxics Assessment. URL: http://www.epa.gov/airtoxics/natamain/. Access Date: 2015 Jan. 29.

[13] Good Guide. Scorecard The Pollution Information Site. URL: http://scorecard.goodguide.com/. Access Date: 2015 Jan. 29.

[14] Commission for Environmental Cooperation. Taking Stock Online. URL: http://www.cec.org/Page.asp?PageID=924&SiteNodeID=1097. Access Date: 2015 Jan. 29.

[15] United States Environmental Protection Agency. 2005 Assessment Results - State Summaries. URL: http://www.epa.gov/ttn/atw/nata2005/tables.html#int. Access Date: 2015 Jan. 29.

[16] Good Guide. Pollution Locator - Toxic Chemical Releases. URL: http://scorecard.goodguide.com/env-releases/us-map.tcl. Access Date: 2015 Jan. 29.

[17] Commission for Environmental Cooperation. Google Earth PRTR Facility Map Layer. URL: http://www.cec.org/Page.asp?PageID=751&SiteNodeID=1120. Access Date: 2015 Jan. 29.

[18] Canadian Environmental Law Association, Environmental Defence. Map Your Community. URL: http://www.pollutionwatch.org/mapsearch.do. Access Date: 2015 Jan. 29.

[19] Setton E, Veerman B, Erickson A, Poplawski K, Cheasley R, Whittaker C et al. CAREX Emissions Mapping Project. URL: http://carexcanada.uvic.ca/emp/. Access Date: 2015 Jan. 29.

[20] Hertwich E, Mateles SF, Pease WS, McKone TE. An Update of the Human Toxicity Potential with Special Consideration of Conventional Air Pollutants. URL: https://www.ntnu.no/c/document_library/get_file?uuid=a76b6602-6052-4a03-a48d-e1d274223eee&groupId=10370. Access Date: 2015 July 24.

[21] Treasury Board of Canada. Federal Contaminated Sites Inventory. URL: http://www.tbs-sct.gc.ca/fcsi-rscf/home-accueil-eng.aspx. Access Date: 2015 July 24.

[22] Environment Canada. National Pollutant Release Inventory and Air Pollutant Emission Summaries and Trends Downloadable Datasets. URL: http://www.ec.gc.ca/inrp-npri/default.asp?lang=en&n=0EC58C98-#Emission_Summaries. Access Date: 2015 Jan. 28.

[23] Environment Canada. National Pollutant Release Inventory (NPRI) Substance List. URL: http://www.ec.gc.ca/inrp-npri/default.asp?lang=En&n=E2BFC2DB-1. Access Date: 2015 July 24.

[24] Environment Canada. Overview of Findings of the National Pollutant Release Inventory (NPRI) Sector Coverage Study for the 2008 Reporting Year. URL: http://www.ec.gc.ca/inrp-npri/default.asp?lang=En&n=615C413A-1. Access Date: 2015 July 24.

[25] Environment Canada. National Pollutant Release Inventory (NPRI) Toolbox. URL: http://www.ec.gc.ca/inrp-npri/default.asp?lang=En&n=65A75CDF-1. Access Date: 2015 July 24.

[26] Environment Canada Pollution Data Division. 1-1-2008. Criteria Air Contaminants Emissions Inventory 2006 Guidebook. Government of Canada. Ottawa.

[27] Statistics Canada (2011). Aircraft Movement Statistics: NAC CANADA Towers and Flight Service Stations: Anual Report (TP 577).

[28] Environment Australia. 3-25-2003. Emissions Estimation Technique Manual for Aggregated Emissions from Aircraft Version 2.2. Commonwealth of Australia. Canberra.

[29] DMTI Spatial. CanMap. URL: http://www.dmtispatial.com/canmap/. Access Date: 2015 Jan. 30.

[30] Railway Association of Canada (2010). Locomotive Emissions Monitoring Program 2010.

[31] Statistics Canada (2010). Canadian Vehicle Survey: Annual 2009 No. 53-233-X.

[32] Environment Australia. 11-22-2000. Emissions Estimation Technique Manual for Aggregated Emissions from Motor Vehicles Version 1.0. Commonwealth of Australia. Canberra.

[33] Statistics Canada. Dissemination block (DB). URL: http://www12.statcan.gc.ca/census-recensement/2011/ref/dict/geo014-eng.cfm. Access Date: 2015 Jan. 30.

[34] Statistics Canada. Population Ecumene file. URL: http://www.statcan.gc.ca/pub/92-159-g/2011001/use-utiliser-eng.htm. Access Date: 2015 July 24.

[35] Canada S (2011). Report on Energy Supply and Demand in Canada 2011 Preliminary No. 57-003-X.

[36] Statistics Canada (2007). Households and the Environment Survey (HES) 2005–2006.

[37] Environment Australia. 11-1-1999. Emissions Estimation Technique Manual for Aggregated Emissions from Domestic Solid Fuel Burning Version 1.0. Commonwealth of Australia. Canberra.

[38] Environment Canada and Hearth Products Association of Canada (2000). Characterization of Organic Compunds from Selected Residential Wood Stoves and Fuels.

[39] Fisher LH, Houck JE, Tiegs PE, McGaughey J (2000). Long-term Performance of EPA-Certified Phase 2 Woodstoves, Klamath Falls and Portland, Oregon: 1998/1999. United States Environmental Protection Agency.

[40] Gullett BK, Touati A, Hays MD (2003). PCDD/F, PCB, HxCBz, PAH, and PM emission factors for fireplace and woodstove combustion in the San Francisco Bay region. Environ Sci Technol.

[41] Hays MD, Smith ND, Kinsey J, Dong Y, Kariher P (2003). Polycyclic aromatic hydrocarbon size distributions in aerosols from appliances of residential wood combustion as determined by direct thermal desorption - GC/MS. J Aerosol Sci.

[42] Houck JE, Crouch J (2002). Updated Emissions Data for Revision of AP-42 Section 1.9, Residential Fireplaces. United States Environmental Protection Agency.

[43] Houck JE, Eagle JE (2006). Task 4 Technical memorandum 2 (Emission Inventory) Control Analysis and Documentation for Residential Wood Combustionin the MANE-VU Region.

[44] Conventional LV, Study WEF (2007). 16th Anual International Emission Inventory Conference.

[45] United States Environmental Protection Agency. Emissions Factors and AP 42, Compilation of Air Pollutant Emission Factors. URL: http://www.epa.gov/ttn/chief/ap42/index.html. Access Date: 2015 Jan. 31.

[46] Statistics Canada. Population by year, province and territory. URL: http://www.statcan.gc.ca/tables-tableaux/sum-som/l01/cst01/demo02a-eng.htm. Access Date: 2015 Jan. 29.

