# Supplementary material for: Identifying potential exposure reduction priorities using regional rankings based on emissions of known and suspected carcinogens to outdoor air in Canada
Source: Environ Health. 2015 Aug 22;14:69. doi: 10.1186/s12940-015-0055-2 (PMC4546257; doi:10.1186/s12940-015-0055-2)
Supplement: Additional file 1: — EMP methods report 2011 final. (PDF 1205 kb) [file 12940_2015_55_MOESM1_ESM.pdf]

CAREX EMISSIONS MAPPING PROJECT

ESTIMATING EMISSIONS OF KNOWN AND SUSPECTED CARCINOGENS TO OUTDOOR AIR - 2011

METHODS FOR:

AIRPLANES TAKING OFF AND LANDING

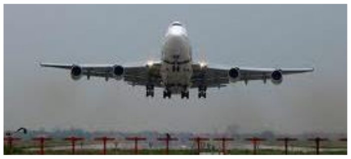

RESIDENTIAL HEATING (GAS, OIL, WOOD)

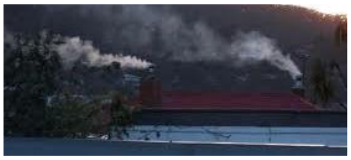

TRAINS

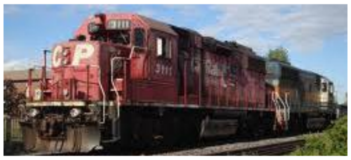

LIGHT AND HEAVY DUTY VEHICLES

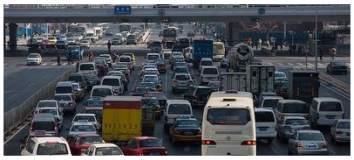

Prepared by:
Eleanor Setton, PhD
Anders Erickson, PhD candidate
CAREX Canada - UNIVERSITY OF VICTORIA SITE

REVISED July 2015

CIHR IRSC
Canadian Institutes of Health Research
Instituts de recherche en santé du Canada

CANADIAN PARTNERSHIP AGAINST CANCER
PARTENARIAT CANADIEN CONTRE LE CANCER

CAREX EMISSIONS PROJECT

TABLE OF CONTENTS

1. INTRODUCTION 2
2. AIRPLANES TAKING OFF AND LANDING 4
3. RESIDENTIAL HEATING (GAS, OIL AND WOOD) 9
4. TRAINS 21
5. LIGHT AND HEAVY DUTY VEHICLES 24

1. INTRODUCTION

The CAREX Emissions Mapping Project was developed with funding from the Canadian Partnership Against Cancer and the Canadian Institutes for Health Research. The main objective is to provide easy access to a wide variety of geospatial data on environmental quality in Canada, as well as to develop an indicator of environmental quality for different regions in Canada. The indicator developed is the sum of total emissions, in toxic equivalents, of known and suspected carcinogens within a region, and is meant to illustrate differences in Canadians' potential exposure to these substances in outdoor air for the year 2011.

Total emissions (in toxic equivalents) was calculated from data reported to the National Pollutant Release Inventory (NPRI) and our own estimates of emissions from airports, residential heating (oil and gas, wood), trains and light duty/heavy duty vehicles. This report provides detailed information on the methods and data used to develop our estimates of emissions.

Our emissions estimates rely on publicly available data on the amount of fuel used or vehicle kilometers travelled, as well as emission factors. Key limitations of our estimates include:

- Only major sources have been incorporated. Other sources may be present, so our estimates may underestimate the total emitted.
- Emission factors were not available for all substances of interest, so the total emissions may be underestimated.
- Emission factors may be uncertain. It is difficult to establish whether emissions are over- or underestimated due to uncertainty in the emission factors.
- Other emissions estimates may use different methods or more detailed data, so our estimates may not agree with others (see Table 1.1).

Table 1.1 Comparison of emission estimates in Canada

Substance	Environment Canada (EC)[1] 2011(kg)	CAREX 2011 (kg)	Comparison	Notes
Cadmium	7,953	26,086	CAREX 3.3 times higher	a
Lead	178,228	169,495	EC 1.05 times higher	b
Benzo[b]fluoranthene	32,641	18,351	EC 1.8 times higher	b
Fine particulates ($PM_{2.5}$)	245,650,000	171,838,236	EC 1.4 times higher	b
Benzo[k]fluoranthene	11,791	5,410	EC 2.2 times higher	c
Benzo[a]pyrene	19,543	8,434	EC 2.3 times higher	c
Indeno(1,2,3-cd)pyrene	14,498	6,254	EC 2.3 times higher	c

a. Environment Canada estimate does not include emissions from vehicle traffic, and the CAREX estimate is higher for residential heating with natural gas and wood, although the emission factors used are those reported in the Environment Canada Criteria Air Contaminants Emissions Inventory 2006 Guidebook[2].
b. Estimates within a factor of 2 are considered to be in reasonable agreement.
c. Additional sources are included in the Environment Canada estimate.

[1] Environment Canada website, Air Pollutant Emission Summaries and Trends. Excel files available for download at http://www.ec.gc.ca/inrp-npri/default.asp?lang=en&n=0EC58C98-. Totals shown do not include open and natural sources.
[2] Environment Canada Criteria Air Contaminants Emissions Inventory 2006 Guidebook (2008). Prepared by the Pollution Data Division of Environment Canada.

2. AIRPLANES TAKING OFF AND LANDING

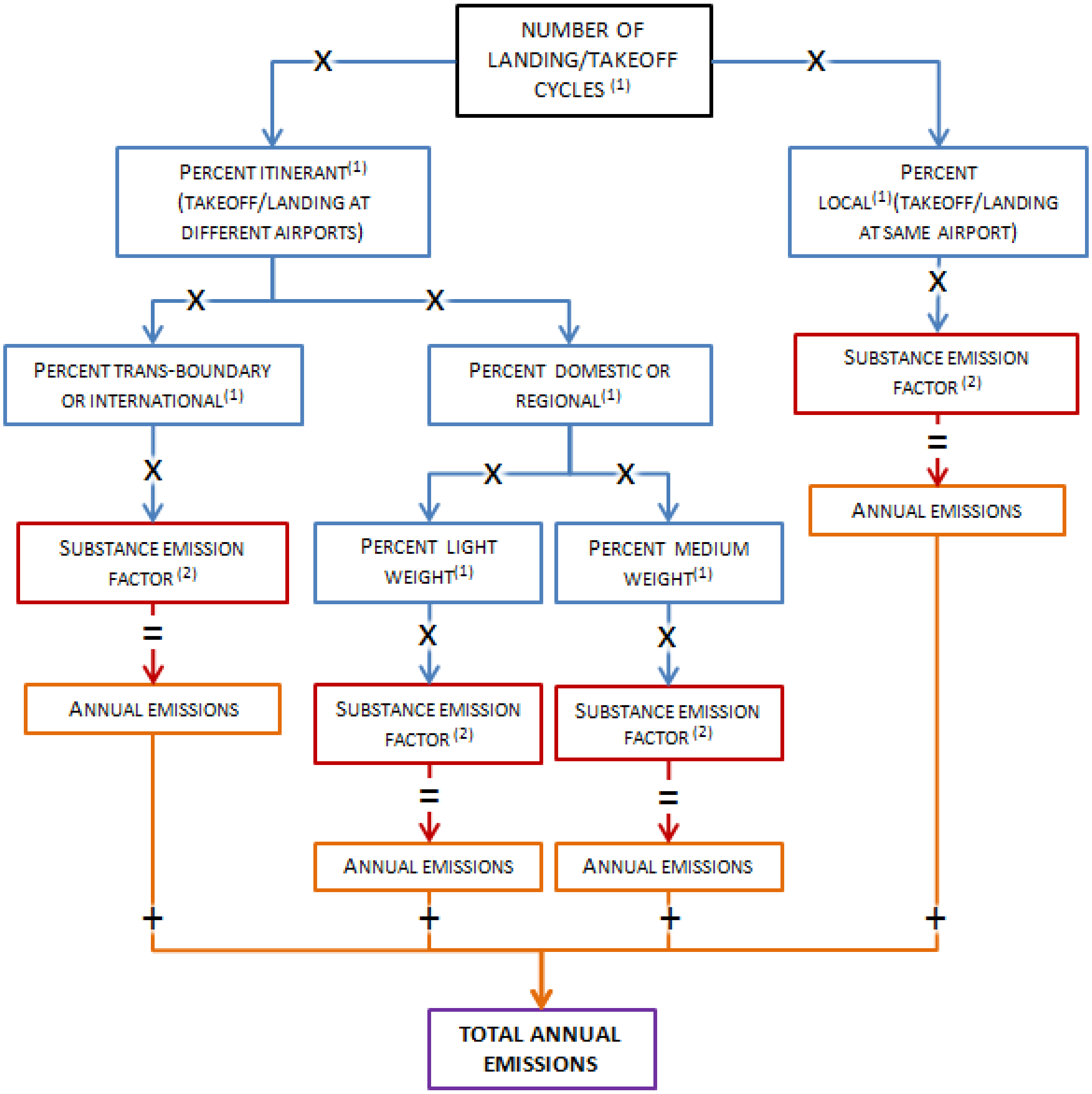

(1) **Statistics Canada. 2011**. *Aircraft Movement Statistics: NAV CANADA Towers and Flight Service Stations: Annual Report (TP 577)*. Catalogue no. 51-209-X.

(2) **Environment Australia. 2003**. Emissions Estimation Technique Manual for Aggregated Emissions from Aircraft.

LIMITATIONS

- These emissions estimates do not account for ground support activities (GSAs). Emissions from GSAs are not expected to contribute significantly to emissions generated from airports as a whole. For example, the proportion of potential emissions missed by not including GSA data was calculated to be an additional 13kg of benzene per year for the Vancouver International Airport (approximately 0.1% of estimated benzene emissions).
- Small airports (n=138) without air traffic control towers or flight service stations were not included in the analysis.
- For ease of calculation, the average proportion of engine type for Canadian airports was used, although airport specific figures are available from Statistics Canada (2011).
- We used emission factors from Australia, published in 2003. We expect these to be reasonably representative of Canadian emission factors.

SPATIAL ACCURACY

- We used the DMTI Spatial Enhanced Points of Interest[3] dataset, which includes most airport locations. Airports not included in the DMTI file were geocoded using Google Earth. Points are located within the airport boundaries, typically on or near runways.

METHODS

The Transportation Division of Statistics Canada has collected and compiled Aircraft Movement Statistics as an annual report for large and medium-sized airports (Statistics Canada 2011). Large airports are those with NAV CANADA control towers and medium airports are those with flight service stations.

Statistics Canada classifies aircraft movements as either 'local' or 'itinerant' movements. Local movements are those in which the aircraft remains in the circuit and lands at the same airport without landing elsewhere. Itinerant movements are where an aircraft proceeds to or arrives from another location; or where an aircraft leaves the circuit (a specified radius of airspace around a control tower, <24km) and returns without landing at another airport. Itinerant movements are further classified as domestic/regional, transboundary, or other international:

- Domestic/regional itinerant movements are flights departing to or arriving from another point in Canada. Aircraft movements are reported on the basis of place "arrived from" or "departed to". This applies to multi-city flights. Therefore, a flight arriving in Toronto from Germany is 'international', but if the same flight carries on to Vancouver, both the departure from Toronto and arrival in Vancouver is considered 'domestic'. Domestic/Regional movements were further divided into two categories, either 'light weight' or 'medium weight', based on the proportion of classified take-off weight groups.

[3] DMTI Spatial (2011) Enhanced Points of Interest (EPOI) User Manual v2011.3.

- Transboundary refers to flights to or from United States including Alaska, Hawaii, and Puerto Rico.
- Other international refers to flights to or from countries outside Canada or the US.

The proportion of itinerant and local aircraft movements at airports with NAV CANADA towers and flight service stations, and the proportion of itinerant aircraft movements classified as domestic/regional , transboundary, or other international are a shown in Table 2.1.

Table 2.1: Proportion of flight types into/out of Canadian airports

Flight Type	NAV Can. Towers	Flight Service Stations
**Itinerant Movements**	**71.4%**	**74.7%**
- Domestic/Regional	80.5%	98.2%
- flight weight proportion (%, light/medium)	(44/56)	(64/36)
- Transboundary	15.3%	1.7%
- Other international	4.2%	0.1%
**Local Movements**	**28.6**	**25.3**

Source: adapted from Statistics Canada (2011)

The predominant method for calculating emissions associated with airports is using Landing/Takeoff (LTO) cycles. A LTO cycle is the sum of all landing and takeoff movements, and incorporates all normal flight and ground operation activities including: descent/approach from a reference height above ground level, touchdown, landing run, taxi in, idle and shutdown, start up and idle, checkout, taxi out, takeoff and climb out to reference height (Environment Australia 2003).

Because each take off and landing is counted in total movements, the airport-specific movement counts provided by Statistics Canada were all divided by two in order to represent one Landing/Takeoff (LTO) cycle. We assume that local movements and movements that leave the circuit but land at the same airport were counted in the same way as itinerant movements (take off and landing equals two movements), and thus were also divided by two to represent one LTO cycle.

Table 2.2 shows the time-weighted aircraft emission factors (kg/LTO) for hydrocarbons and total suspended particulates by flight type. Emission factors for each 'mode' in a LTO have been weighted by the relative time spent in each LTO mode according to the different flight types. For example, International flights spend 78% of its LTO taxiing compared to 60% for General Aviation. International flights were further weighted by plane type (turbo prop versus jumbo). Hydrocarbons (HC) and Total Suspended Particles (TSP) were summed across LTO modes for each flight types (International, Domestic, Regional, and General Aviation).

The proportion of “Light” flights was assigned the emission factor for “Regional” in Table2.3. “Domestic” flights were assigned the emission factor for “Domestic” in Table 2.3. The proportion of International and transboundary flights were combined and assigned the “International” emission factor in Table 2.3. Local movements are flights that take-off and land at the same airport and were assigned the “General Aviation (Local) emission factor in Table 2.2.

Table 2.2: Time-Weighted Emission Factors by LTO Mode (kg/LTO)

Flight Types	HC	TSP	Flight Type Definitions p.5-6 (NPI, 2003)
International			Transnational flights, including to the U.S.
Approach*	0.0326	0.0301	
Taxi*	2.4016	0.5844	
Take-off*	0.0021	0.0013	
Climb out*	0.0061	0.0062	
**LTO Sum***	**2.4424**	**0.6220**	
Domestic			Flights between two major airports within the country – use of high capacity jets (>38 seats or >4,200kg payload)
Approach	0.0050	0.0124	
Taxi	0.6950	0.0905	
Take-off	0.0001	0.0004	
Climb out	0.0013	0.0033	
**LTO Sum**	**0.7014**	**0.1066**	
Regional			Flights linking smaller rural centres with principle cities.
Approach	0.0176	0.0093	
Taxi	2.9640	0.1646	
Take-off	0.0001	0.0003	
Climb out	0.0010	0.0041	
**LTO Sum**	**2.9827**	**0.1783**	
Gen. Aviation (Local)			Includes private, business, training, agriculture, charter, recreational sport.
Approach	0.0095	0.0726	
Taxi	0.0453	0.5192	
Take-off	0.00003	0.0002	
Climb out	0.0063	0.0495	
**LTO Sum**	**0.0611**	**0.6415**	

Source: modified from Table 2 & 3 in Environment Australia (2003)

* calculated using average time per LTO mode between Jumbo and Turboprop planes.

Once total hydrocarbons and total suspended particulate amounts are estimated, the factors listed in Table 2.3 were used to estimate emissions for specific substances. With respect to TSP, there are two major engine types, gas turbine (jet) and reciprocating piston (internal combustion). TSP weight fractions for ‘Jet’ were used in place where *no data* were available for ‘Piston’ type aircrafts. For ease of calculation, the average proportion of engine type for Canadian airports was used, although airport specific figures are available from Statistics Canada (2011).

Table 2.3: Weight Fractions for Volatile Organic Compound & Total Suspended Particles Species

Substance	VOC Speciation Weight Fractions	TSP Speciation Weight Fractions	
	Commercial	Piston	Jet
1,3-butadiene	0.018	-	-
Acetaldehyde	0.047	-	-
Benzene	0.019	-	-
Ethylbenzene	0.002	-	-
Formaldehyde	0.141	-	-
PAH compounds	0.0106	-	-
Styrene	0.004	-	-
Arsenic	-	no data	0.0053
Cadmium	-	no data	0.0005
Chromium III	-	0.00035	0.0037
Chromium IV	-	0.00015	0.0016
Lead	-	no data	0.0055
Nickel	-	0.0005	0.0055
PM_{10}	-	0.9	0.976

Source: pg 10-11 and 14-15, Environment Australia (2003)

3. RESIDENTIAL HEATING (GAS, OIL AND WOOD)

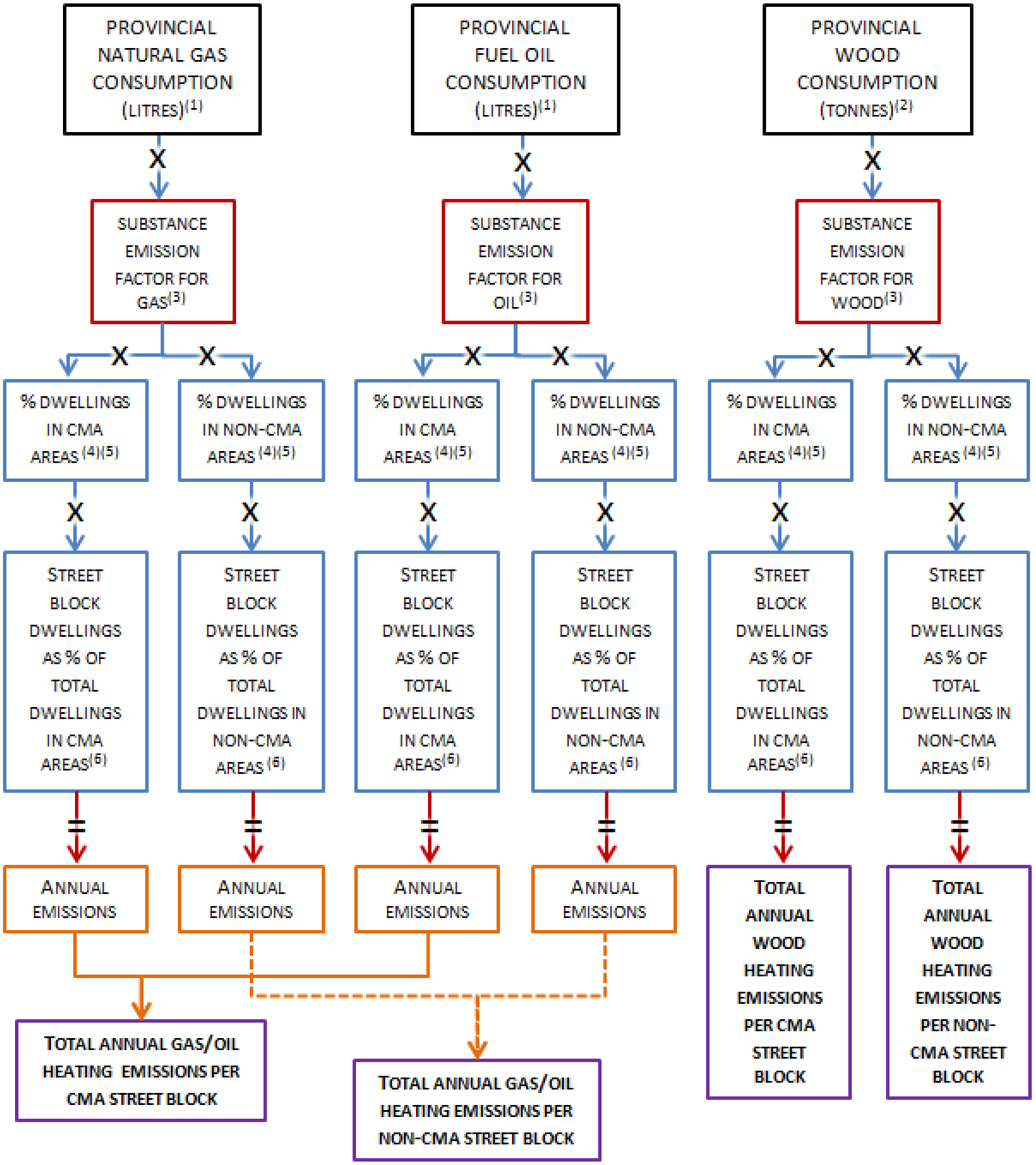

(1) **Statistics Canada. 2011**. Report on Energy Supply and Demand 2011, includes natural gas and gas plant liquids, and kerosene, stove oil, light fuel oil and heavy fuel oil.
(2) **Environment Canada. 2008**. Criteria Air Contaminants Emissions Inventory 2006 Guidebook.
(3) Emission factors from various sources (see Tables 3.5 – 3.8).
(4) **Statistics Canada. 2006**. Households and the Environment Survey.
(5) Statistics Canada 2011 Census Subdivision boundary file
(6) Statistics Canada 2011 Street Block point file

LIMITATIONS

- Provincial estimates are allocated evenly among all residential dwellings in each area (provincial CMA and non-CMA regions). This may over or underestimate emissions in smaller areas, if the proportions of gas/oil or wood burning are different than the provincial proportions.
- There is a lot of uncertainty about the accuracy of emission factors. We use the same factors across Canada so relative comparisons can be made among regions, but we do not know if emissions are being over or underestimated.

SPATIAL ACCURACY

Street block centroid points are produced by Statistics Canada, along with the following descriptions[4]:

- Households are linked to block-face representative points when the street and address information is available; otherwise, they are linked to dissemination block (DB) representative points.
- A block-face is one side of a street between two consecutive features intersecting that street. The features can be other streets or boundaries of standard geographic areas. Block-faces are used for generating block-face representative points, which in turn are used for geocoding and census data extraction when the street and address information are available.
- A dissemination block (DB) is an area bounded on all sides by roads and/or boundaries of standard geographic areas. The dissemination block is the smallest geographic area for which population and dwelling counts are disseminated. Dissemination blocks cover all the territory of Canada.

Typically, in densely populated areas, individual residential locations are very close to the street block centroid (+/- a few hundred metres). In rural or remote areas, homes may be much further away from the street block centroid.

METHODS

Consumption of petroleum-based fuels, by province for 2011, from the Report on Energy Supply and Demand in Canada were used to determine total provincial fuel consumption for natural gas (including gas plant liquids) and oil (including kerosene, stove oil, light fuel oil and heavy fuel oil)(Table 3.1). Data were reported for Nunavut, Northwest Territories and Yukon as one value. These were split proportionally based on reported populations for each jurisdiction, according to population reported by Statistics Canada for 2011.

[4] GeoSuite, Reference Guide Census year 2011, Statistics Canada.

Consumption of wood by appliance type by province (Table 3.2) was derived from a survey commissioned by Environment Canada in 2006 (Canadian Facts of Toronto, 9,588 responses,) as reported in the Criteria Air Contaminants Emissions Inventory 2006 Guidebook pg. 117 (Environment Canada 2008). Data were reported for Nunavut and Northwest Territories as one value; this was split proportionally based on reported populations for each jurisdiction, according to population reported by Statistics Canada for 2011.

The Statistics Canada Households and the Environment Study (Statistics Canada 2006(b)) provided responses from 28, 334 individuals across Canada. Respondents were identified by province and selected Census Metropolitan Areas (CMAs), and type of main heating equipment (Table 3.3). These data were used to calculate the percentage of respondents using each heating type in CMAs and Non-CMAs for each province (Table 3.4), using the individual population weight included in the survey. No survey responses were available for Nunavut, the Northwest Territories, or Yukon. We assumed no difference between urban and rural dwellers in these jurisdictions in subsequent steps. Similarly, no CMA was identified for PEI, so we assumed all residents were included in Non-CMA areas.

Census subdivision boundaries from Statistics Canada provided digital boundaries for census subdivisions (CSDs) in Canada for the 2011 Census. The file also includes the Census Metropolitan Area (CMA) name for any census subdivisions within a CMA, and the total number of dwellings in each CSD. This file was used to identify the number and percent of dwellings per province within and outside of CMAs listed in the Statistics Canada Households and the Environment Survey, and our preliminary emission estimates were calculated for the CSDs.

Block point centroids, Statistics Canada (includes CSD identifier for each block point).
This is a geographic information system file, providing point locations for 493,192 street block centroids, and includes dwelling counts for each point. We used this file to identify the number and percent of block points within each CSD and allocated the preliminary emission estimates to each block point.

Emission factors for most substances were obtained from a variety of sources and are listed in Tables 3.5, 3.6 and 3.7. Emission factor references are listed in Table 3.8. Emissions of fine particulates ($PM_{2.5}$) from use of heavy fuel oil were estimated based on the typical sulfur content of the oil (Table 3.9).

Table 3.1. Amount of petroleum-based fuel consumed, by province

	Annual Consumption					
	total coal (kilo tonnes)	natural gas (gigalitres)	gas plant liquids* (megalitres)	kerosene and stove oil (megalitres)	light fuel oil (megalitres)	heavy fuel oil (megalitres)
NU				0		
NT		5.2	5.9		26.7	
YK			1.9	5.1	3.8	
BC		2,171.7	35.2	0.1	22.2	
AB	12.0	4,401.6	66.5	1.1	0.2	
SK	86.0	897.8	12.7	7.5	1.9	
MB		523.3	8.9		4.7	
ON		9,073.2	421.4	11.5	589.6	
PQ		600.8	39.7	94.5	602.5	
NB		15	9.5	0.4	239.1	39.2
NS		3.6	33.6	3.2	635.9	0.4
PE			5.2	0.3	149.5	
NL			8.8	0.4	163.4	

*Gas plant liquids includes propane, butane and ethane

Source: Statistics Canada. 2011. Report on Energy Supply and Demand in Canada

Note: Electricity used primarily in Nunavut

Note: Natural gas and gas plant liquids considered as ‘natural gas’ ; kerosene, stove oil, light fuel oil and heavy fuel oil considered as ‘oil’, for application of percents listed in Table 3.4.

Table 3.2. Amount of wood burned by appliance type and province

Appliance Type	Annual Tonnes Burned												
	NL	PE	NS	NB	PQ	ON	MB	SK	AB	BC	YT	NT	NU
Fireplace, open, no doors	3,192	965	13,429	9,187	228,685	163,694	12,139	8,685	59,396	109,868	144	199	117
Fireplace, glass doors	3,837	1,502	11,413	9,337	337,664	188,750	14,076	18,275	53,676	79,570	225	309	181
Fireplace, conventional insert	645	0	6,735	4,950	47,052	77,149	3,808	2,445	5,977	49,272	64	88	52
Fireplace, advance technology insert	1,392	0	0	0	24,910	17,474	0	0	4,599	6,375	8	11	7
Fireplace, advanced technology	0	1,234	3,952	2,775	68,848	14,177	1,090	2,254	5,557	7,500	81	111	65
Stove, not air tight	64,728	7,487	47,707	46,684	431,421	216,774	32,773	16,568	35,932	145,415	1,122	1,539	904
Stove, air tight	95,326	18,109	151,309	141,141	1,211,232	586,363	44,115	39,861	41,138	217,336	2,713	3,723	2,187
Stove, advanced technology	15,350	4,117	10,929	12,187	344,584	77,808	10,805	9,870	17,230	39,447	367	504	296
Furnace or Boiler	155,129	22,630	152,196	146,240	736,219	276,120	41,056	28,756	5,206	73,945	3,390	4,653	2,732
Other equipment	0	555	5,606	2,475	29,061	30,167	2,864	637	4,763	21,224	83	114	67

Source: Environment Canada. 2008. Criteria Air Contaminants Emissions Inventory 2006 Guidebook

Table 3.3. CMA codes and heating equipment codes in the Households and the Environment Survey

CMA Codes:

01	NL - St. John's CMA
02	NL – Non CMA
03	PE (no CMA identified)
04	NS – Halifax CMA
05	NS – Non CMA
06	NB – Saint John CMA
07	NB – Non CMA
08	PQ – Saugenay – Chicoutimi CMA
09	PQ – Gatineau CMA
10	PQ – Montreal CMA
11	PQ – Quebec City CMA
12	PQ – Sherbrooke CMA
13	PQ – Trois-Rivieres CMA
14	PQ – Non CMA
15	ON – Hamilton CMA
16	ON – Kingston CMA
17	ON – Kitchener-Waterloo CMA
18	ON – London CMA
19	ON – Oshawa CMA
20	ON – Ottawa CMA
21	ON – St. Catherines-Niagara CMA
22	ON – Sudbury CMA
23	ON – Thunder Bay CMA
24	ON – Toronto CMA
25	ON – Windsor CMA
26	ON – Non CMA
27	MB – Winnipeg CMA
28	MB – Non CMA
29	SK – Regina CMA
30	SK – Saskatoon CMA
31	SK – Non CMA
32	AB – Calgary CMA
33	AB – Edmonton CMA
34	AB – Non CMA
35	BC – Abbotsford CMA
36	BC – Vancouver CMA
37	BC – Victoria CMA
38	BC – Non CMA

Heating Type Codes:

01	forced air natural gas furnace
02	forced air oil furnace
03	forced air electric furnace
04	forced air hot water system
05	hot water radiators
06	electric baseboards
07	other electric heating
08	wood stove or fireplace
09	other
97	don't know
98	refusal

Source: Statistics Canada. 2006. Households and the Environment Survey.

Table 3.4. Percent of residents by heating type, based on province and CMA/NON-CMA status

		Percent		
		Furnace natural gas	Furnace oil	Wood
CMA	BC	64.5	43.8	13.0
NONCMA		35.5	56.2	87.0
CMA	AB	63.7	54.7	11.3
NONCMA		36.3	45.3	88.7
CMA	SK	48.1	3.0	5.3
NONCMA		51.9	97.0	94.7
CMA	MB	79.4	0.0	4.6
NONCMA		20.6	100.0	95.4
CMA	ON	78.0	40.9	12.4
NONCMA		22.0	59.1	87.6
CMA	PQ	89.6	68.5	18.1
NONCMA		10.4	31.5	81.9
CMA	NB	24.4	18.9	5.5
NONCMA		75.6	81.1	94.5
CMA	NS	59.2	38.4	9.0
NONCMA		40.8	61.6	91.0
CMA	PE	0.0	0.0	0.0
NONCMA		100.0	100.0	100.0
CMA	NL	100.0	32.0	4.5
NONCMA		0.0	68.0	95.5

Source: Statistics Canada. 2006. Households and the Environment Survey.

Table 3.5. Emission factors for lead, cadmium, arsenic, hexavalent chromium, nickel, and fine particulates

Type of fuel/appliance	Emission Factor Units	**Lead** Emission Factor	Reference	**Cadmium** Emission Factor	Reference	**Arsenic** Emission Factor	Reference	**Hexavalent Chromium** Emission Factor	Reference	**Nickel** Emission Factor	Reference	**Fine particultes** (PM2.5) Emission Factor	Reference
natural gas	kg/gigalitre	8.01E-03	a	1.76E-02	a	1.56E-02	b			3.36E-02	b	1.22E+02	a
gas plant liquid	kg/kilolitre	1.15E+00	a	1.43E-02	a							5.50E-02	a
kerosene	kg/kilolitre	7.29E-05	a	3.89E-05	a							1.00E-01	a
light oil	kg/kilolitre	7.28E-05	a	3.89E-05	a							1.00E-01	a
heavy oil	kg/kilolitre	1.81E-04	a	4.78E-05	a							See Table 9	
wood - open fireplace	kg/tonne	3.01E-04	c	9.40E-04	c	7.52E-05	c					1.84E+01	a
wood - fireplace with glass doors	kg/tonne	3.01E-04	c	9.40E-04	c	7.52E-05	c					1.29E+01	a
wood - conventional insert	kg/tonne	1.93E-04	c	1.10E-05	c	4.81E-05	c	1.47E-07	c	7.00E-06	c	1.36E+01	a
wood - advanced technology insert	kg/tonne	1.93E-04	c	1.10E-05	c	4.81E-05	c	1.47E-07	c	7.00E-06	c	4.80E+00	a
wood - advanced technology fireplace	kg/tonne	1.93E-04	c	1.10E-05	c	4.81E-05	c	1.47E-07	c	7.00E-06	c	4.80E+00	a
wood - stove, not air tight	kg/tonne	1.93E-04	c	1.10E-05	c	4.81E-05	c	1.47E-07	c	7.00E-06	c	2.32E+01	a
wood - stove, air tight	kg/tonne	1.93E-04	c	1.10E-05	c	4.81E-05	c	1.47E-07	c	7.00E-06	c	1.36E+01	a
wood - advanced technology stove	kg/tonne	8.83E-05	c	1.00E-05	c	2.21E-05	c	1.47E-07	c	1.00E-05	c	4.80E+00	a
wood - furnace boiler	kg/tonne	8.83E-05	c	1.00E-05	c	2.21E-05	c	1.47E-07	c	1.00E-05	c	1.33E+01	a
wood - other	kg/tonne	8.83E-05	c	1.00E-05	c	2.21E-05	c	1.47E-07	c	1.00E-05	c	1.36E+01	a

See emission factor references listed in Table 3.8.

Table 3.6. Emission factors for acetaldehyde, benzene, 1,3-butadiene, formaldehyde and dioxin/furan

Type of fuel/appliance	Emission Factor Units	**Acetaldehyde** Emission Factor	Reference	**Benzene** Emission Factor	Reference	**1,3-butadiene** Emission Factor	Reference	**Formaldehyde** Emission Factor	Reference	**Dioxin/Furan** Emission Factor	Reference
natural gas	kg/gigalitre			3.36E-02	b			3.40E-01			
gas plant liquid	kg/kilolitre										
kerosene	kg/kilolitre									1.50E-10	a
light oil	kg/kilolitre									1.50E-10	a
heavy oil	kg/kilolitre									1.50E-10	a
wood - open fireplace	kg/tonne	5.30E-01	j	3.00E-01	g	3.60E-01	c	1.20E+00	g	5.00E-10	a
wood - fireplace with glass doors	kg/tonne	3.10E-01	j	3.00E-01	g	3.60E-01	c	1.20E+00	g	5.00E-10	a
wood - conventional insert	kg/tonne	3.10E-01	j	5.72E-01	d	1.21E-01	e	9.70E-01	f	5.00E-10	a
wood - advanced technology insert	kg/tonne	3.10E-01	j	5.72E-01	d	1.21E-01	e	9.70E-01	f	5.00E-10	a
wood - advanced technology fireplace	kg/tonne	3.10E-01	j	5.72E-01	d	1.21E-01	e	9.70E-01	f	5.00E-10	a
wood - stove, not air tight	kg/tonne	5.30E-01	j	5.72E-01	d	1.21E-01	e	9.70E-01	f	5.00E-10	a
wood - stove, air tight	kg/tonne	3.10E-01	j	5.72E-01	d	1.21E-01	e	9.70E-01	f	5.00E-10	a
wood - advanced technology stove	kg/tonne	3.10E-01	j	1.74E-01	e	7.00E-03	e	5.00E-01	c	5.00E-10	a
wood - furnace boiler	kg/tonne	3.10E-01	j	1.74E-01	e	7.00E-03	e	5.00E-01	c	5.00E-10	a
wood - other	kg/tonne	3.10E-01	j	1.74E-01	e	7.00E-03	e	5.00E-01	c	5.00E-10	a

See emission factor references listed in Table 3.8.

Table 3.7. Emission factors for polycyclic aromatic hydrocarbons

Type of fuel/appliance	Emission Factor Units	**Benzo[a]pyrene** Emission Factor	Reference	**Benzo[b]fluoranthene** Emission Factor	Reference	**Benzo[k]fluoranthene** Emission Factor	Reference	**Benzo[a]anthracene** Emission Factor	Reference	**Chrysene** Emission Factor	Reference	**Indeno(1,2,3-cd)pyrene** Emission Factor	Reference
natural gas	kg/gigalitre	1.92E-05	a	2.88E-05	a	2.88E-05	a	2.92E-05	b			2.88E-05	a
gas plant liquid	kg/kilolitre												
kerosene	kg/kilolitre	1.61E-07	a	1.78E-07	a	1.78E-07	a					2.57E-07	a
light oil	kg/kilolitre	1.61E-07	a	1.78E-07	a	1.78E-07	a					2.57E-07	a
heavy oil	kg/kilolitre			1.78E-07	a	1.78E-07	a					2.57E-07	a
wood - open fireplace	kg/tonne	5.20E-04	i	2.71E-03	b	3.85E-04	i	5.55E-04	i	5.32E-03	b	3.05E-04	i
wood - fireplace with glass doors	kg/tonne	5.20E-04	i	2.71E-03	b	3.85E-04	i	5.55E-04	i	5.32E-03	b	3.05E-04	i
wood - conventional insert	kg/tonne	7.28E-04	e	9.90E-04	e	1.80E-04	e	4.51E-04	b	8.40E-04	e	4.30E-04	e
wood - advanced technology insert	kg/tonne	7.28E-04	e	9.90E-04	e	1.80E-04	e	4.51E-04	b	8.40E-04	e	4.30E-04	e
wood - advanced technology fireplace	kg/tonne	7.28E-04	e	9.90E-04	e	1.80E-04	e	4.51E-04	b	8.40E-04	e	4.30E-04	e
wood - stove, not air tight	kg/tonne	7.28E-04	e	9.90E-04	e	1.80E-04	e	4.51E-04	b	8.40E-04	e	4.30E-04	e
wood - stove, air tight	kg/tonne	7.28E-04	e	9.90E-04	e	1.80E-04	e	4.51E-04	b	8.40E-04	e	4.30E-04	e
wood - advanced technology stove	kg/tonne	3.20E-05	h	7.70E-05	h	1.40E-05	h	6.30E-05	h	7.30E-05	h	4.30E-04	e
wood - furnace boiler	kg/tonne	3.20E-05	h	7.70E-05	h	1.40E-05	h	6.30E-05	h	7.30E-05	h	4.30E-04	e
wood - other	kg/tonne	3.20E-05	h	7.70E-05	h	1.40E-05	h	6.30E-05	h	7.30E-05	h	4.30E-04	e

Note – Benzo[a]anthracene and chrysene were classified as “No Emission Factor” for Oil and Gas estimate due to few or no factors available. See emission factor references listed in Table 3.8.

Table 3.8. Emission Factor References

Code	Reference
a	"Criteria Air Contaminants Emissions Inventory Guidebook" (2008), prepared by the Pollution Data Division of Environment Canada
b	US Environmental Protection Agency AP-42 database
c	Emissions Estimation Technique Manual for Aggregated Emissions from Domestic Solid Fuel Burning (1999), Environment Australia.
d	Average of values from e and f
e	Characterization of Organic Compounds from Selected residential Wood Stoves and Fuels (2000), prepared by Environment Canada in collaboration with the Hearth Products Association of Canada
f	Conventional Woodstove Emission Factor Study (2007), by Victor Li of Environment Canada, presented in Session 5 of the 16th Annual International Emission Inventory Conference in Raleigh, NC.
g	Updated Emissions Data for Revision of AP-42 Section 1.9, Residential Fireplaces (2002), prepared by JE Houck and J Crouch for the US Environmental Protection Agency
h	Long-Term Performance of EPA-Certified Phase 2 Woodstoves, Klamath Falls and Portland, Oregon: 1998/1999 (2000), prepared by LH Fisher, JE Houck, PE Tiegs and J McGaughey for the US Environmental Protection Agency
i	Average of values from:<br>PCDD/F, PCB, HxCBz, PAH, and PM Emission Factors for Fireplace and Woodstove Combustion in the San Francisco Bay Region (2003) by BK Gullet, A Touati, and MD Hays in Environmental Science and Technology Volume 37, No. 9, pp 1758-1765<br>and<br>Polycyclic aromatic hydrocarbon size distribution in aerosols from appliances of residential wood combustion as determined by direct thermal desorption-GC/MS (2003), by MD Hays, ND Smith, J Kinsey, Y Dong and P Kariher, in Journal of Aerosol Science, Volume 34, pp 1061-1084.
j	Task 4 Technical Memorandum 2 (Emission Inventory) Control Analysis and Documentation for Residential Wood Combustion in the MANE-VU Region (2006), by JE Houck and BN Eagle of OMNI Environmental Services, for the Mid-Atlantic Regional Air Management Association.

Table 3.9. Emission factors for fine particulates from heavy fuel oil

Province	Sulfur content (percent)	Emission factor* (kg/kilolitre)
NL	1.97	0.592572
PE	1.97	0.592572
NS	1.97	0.592572
NB	1.97	0.592572
PQ	1.07	0.360732
ON	1.76	0.538476
MB	1.56	0.486956
SK	1.56	0.486956
AB	1.56	0.486956
BC	1.56	0.486956
YT	1.56	0.486956
NT	1.56	0.486956
NU	1.56	0.486956

* Emission factor = 0.23*((1.12*sulfur content) +0.37)
from Environment Canada (2008) Criteria Air Contaminants Emissions Inventory Guidebook, page 103-4.

4. TRAINS

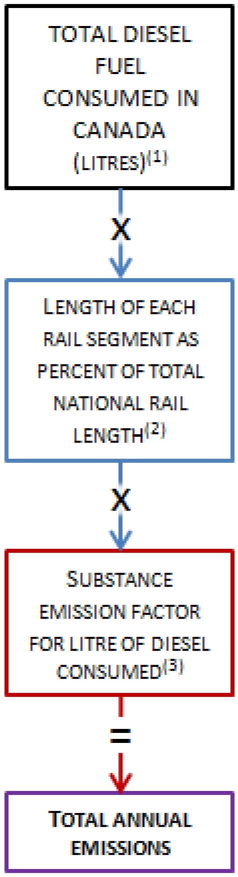

(1) **RAC (Railway Association of Canada). 2010**. Locomotive Emissions Monitoring Program 2010. Ottawa Ontario.
(2) **DMTI Spatial CanMap® Streetfiles** , via University of Victoria Library under the Data Liberations Initiative. (http://www.dmtispatial.com/Software-And-Data/CanMap.html)
(3) **Environment Australia. 1999**. Emissions Estimation Technique Manual for Aggregated Emissions from Railways.

LIMITATIONS

- These estimates do not include activities in rail yards, which are a local source of emissions. It has been estimated by the Railway Association of Canada that in 2006, freight and passenger trains accounted for approximately 97 percent of total fuel consumed. Activities in rail yards accounted for the remaining 3 percent of total fuel consumed[5].
- We do not have any information on volume of train traffic for different rail segments. A kilometre of rail near a major port is assigned the same level of emissions as a kilometre of rail in a remote rural area. This could underestimate emissions where rail volume is high, and overestimate emissions where rail traffic is low.
- We used emission factors from Australia, circa 1999. These may be outdated. For particulate matter, the Australian emission factor is 1.39 grams per litre of fuel consumed compared to the RAC (2006) emission factor of 1.24 grams per litre of fuel consumed for freight trains and 1.27 grams per litre of fuel consumed for passenger trains. Our estimates may therefore be a slight overestimates, in the range of 1 percent.

SPATIAL ACCURACY

- We used DMTI Spatial CanMap Streetfiles[6] file for 2011, which includes CanMap Rail to identify operational railways (codes 961, 962 and 963) in Canada. Typically, the positional accuracy of the DMTI railways is +/- 30 metres.

METHODS

The total amount of diesel fuel consumed for rail transportation (freight and passenger) in Canada in 2010 was reported to be 2,006,050,000 litres in RAC (2010). Total litres consumed were allocated to each rail segment based on segment length as a percentage of total kilometers of rail in Canada. We used the DMTI Spatial file of railways in Canada, and selected only those segments listed as 'Operational' to calculate total national length and segment length as a percentage of total national length.

Emission factors based on litres of fuel consumed (Table 4.1) were then used to estimate total emissions per segment.

---

[5] **RAC (Railway Association of Canada). 2010**. Locomotive Emissions Monitoring Program 2010. Ottawa Ontario. Page 7.
[6]) **DMTI Spatial CanMap® Streetfiles. 2011.** http://www.dmtispatial.com/Software-And-Data/CanMap.html

Table 4.1. Emission factors per litre of diesel fuel consumed

Substance	Emission Factor (grams per litre)
Acetaldehyde	0.0755
Arsenic and compounds	0.00000417
Benzene	0.044
1,3-Butadiene	0.0401
Cadmium and compounds	0.0000931
Hexavalent Chromium	0.00000367
Ethylbenzene	0.00152
Formaldehyde	0.223
Lead and compounds	0.0000417
Nickel and compounds	0.0000208
Particulate matter ≤ 2.5 microns*	1.39
Polycyclic aromatic compounds	0.0188

Source: Environment Australia. 1999. Emissions Estimation Technique Manual for Aggregated Emissions from Railways, Environment Australia, 1999.

* The emission factor listed is reported for PM_{10} in the Environment Australia manual; however, we use it for $PM_{2.5}$ based on the following: "With the advent of improved combustion and exhaust filtration techniques eliminating the visual soot that traditionally characterized diesel engines, the particulate matter that is now predominant is in the 2.5 microns and below size range." [7]RAC 2006, pg. iv.

[7] **RAC (Railway Association of Canada). 2006**. Locomotive Emissions Monitoring Program 2006. Ottawa Ontario. Page iv.

5. LIGHT AND HEAVY DUTY VEHICLES

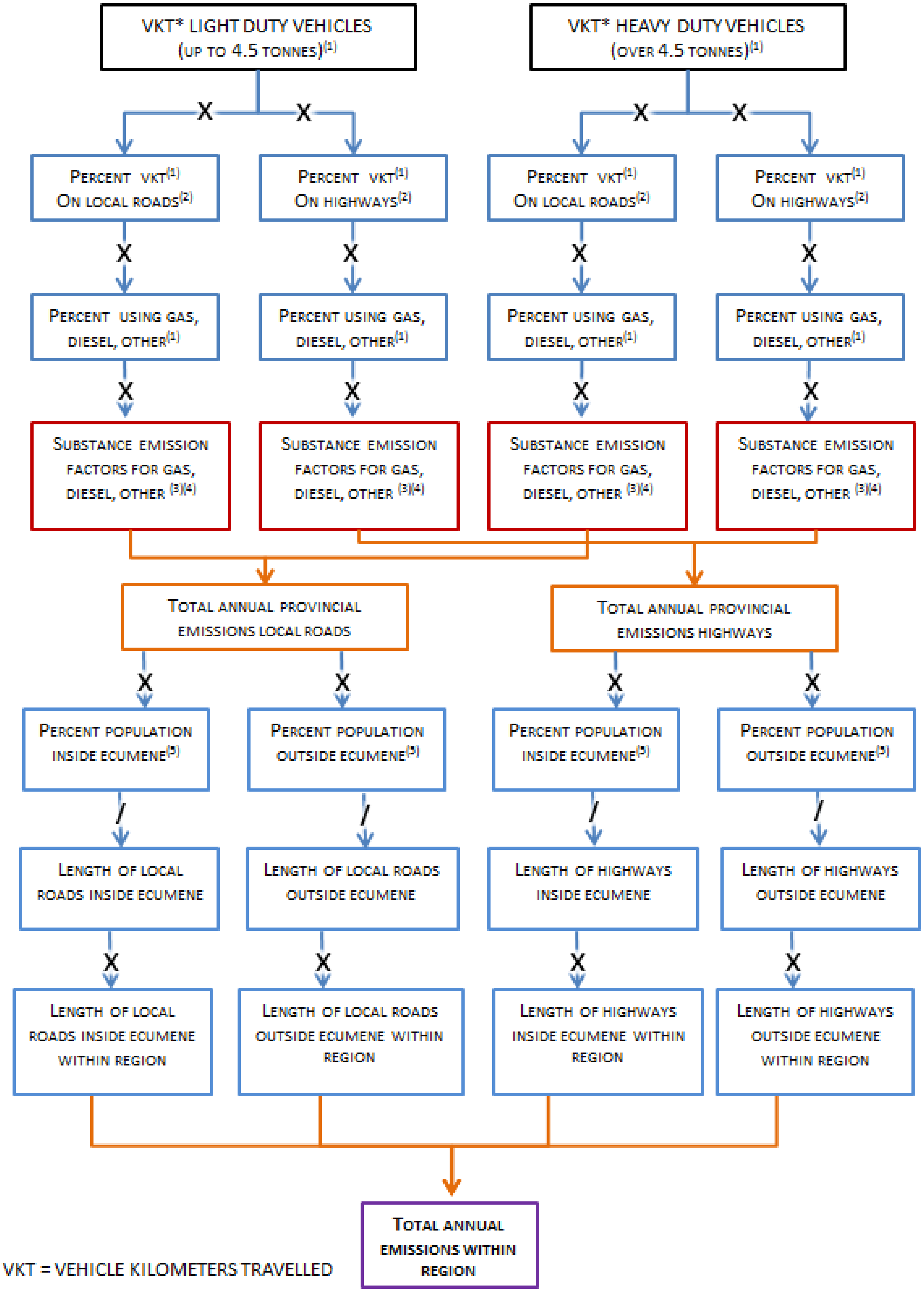

* VKT = VEHICLE KILOMETERS TRAVELLED

(1) **Statistics Canada. 2009**. Canadian Vehicle Survey: Annual 2009 (catalogue no. 53-223-X)
(2) **DMTI Spatial CanMap® Streetfiles** 2011, via University of Victoria Library under the Data Liberations Initiative. (http://www.dmtispatial.com/Software-And-Data/CanMap.html)
(3) **Environment Australia. 2000**. Emissions Estimation Technique Manual for Aggregated Emissions from Motor Vehicles – Version 1.0
(4) Speciation factors for diesel fine particulates based on literature review, see Table 5.11.
(5)**Statistics Canada. 2011.** Population ecumene file (http://www.statcan.gc.ca/pub/92-159-g/2011001/use-utiliser-eng.htm)

LIMITATIONS:

- We did not consider vehicle age – older cars emit more, newer cars less.
- We did not include emissions from off road vehicles.
- Emission factors are from Australia. Emissions are expected to be similar in Canada, as vehicles and fuels are similar. The Pollution Data Division of Environment Canada has estimated vehicle emissions for 2006, but the emission factors employed were not available for our estimate.
- Emission factors are relatively old ~ 2000. We expect that newer vehicles may emit less, so the estimates may be higher than actual for 2011.
- We recognize that there is more traffic on urban roads than rural roads, but did not have any data on traffic volume by region. The estimates allocate emissions based on road length only, so local roads of the same length in urban areas and rural areas will be assigned the same amount of emissions. This may underestimate emissions in urban areas, and overestimate emissions in rural areas.

SPATIAL ACCURACY

- We used the CanMap Streetfiles dataset from DMTI Spatial, which includes an up-to-date road network. Typically, the positional accuracy of the DMTI roads is +/- 30 metres.

METHODS

Annual vehicle kilometers travelled (VKT) are provided by vehicle type by province (Table 5.1); by vehicle and road type nationally (Table 5.2), and by vehicle and fuel type nationally (Table 5.3), based on Statistics Canada Canadian Vehicle Survey: Annual 2009. This survey includes responses from owners of a sample of 26,997 vehicles in the provinces and additional sample of 16,488 vehicles in the territories. Response rates (via telephone interviews) varied from 45 percent to 60 percent in the provinces, but were much lower in Yukon (15 percent), the Northwest Territories (14 percent) and Nunavut (7 percent) as these areas were surveyed by mail only.

We used emission and speciation[8] factors reported in Environment Australia (2000) for estimating vehicle emissions per VKT of total VOCs (Table 5. 4), PM_{10} (Table 5.5), lead (assuming unleaded gas)(Table 5.6), acetaldehyde, benzene, 1,3-butadiene, ethylbenzene, and formaldehyde (Table 5.7). Speciation factors for PM_{10} are provided for cadmium, hexavalent chromium, lead (from diesel and other fuel exhaust), and nickel (Table 5.8). In general, Environment Australia developed emission factors using US EPA models MOBILE5a and PART5. Emission factors for Canada have been developed using the MOBILE6.2C model, but were not publicly available. Emission and speciation factors for diesel fine particulates (diesel $PM_{2.5}$) were derived based on a literature review conducted by CAREX staff (Table 5.9).

We then allocated the total emission for each substance based on VKT for local roads and highways to areas inside and outside the national population ecumene[9] (all areas with a population density of 0.4 persons or more per square kilometer), in proportion to the population present inside and outside the ecumene as per the Statistics Canada Block file[10](Table 5.10).

Finally, we derived emissions per kilometer for each road type (local and highway) for areas inside and outside the ecumene in all provinces and territories by dividing the allocated emissions by the road length within each area.

Roads were categorized by DMTI Spatial as:

1 Expressway
2 Primary Highway
3 Secondary Highway
4 Major Road
5 Local Road
6 Trail – not used for analysis

The length in kilometers of different road types by province was calculated using the DMTI Spatial file. Road categories 1, 2 and 3 were combined to represent “Roads with 80km speed limit or more” (to match the Canadian Vehicle Survey), and also called ‘Highway’ (to match available emission factors). Road categories 4 and 5 were combined to “All other roads” (to match the Canadian Vehicle Survey), and also called ‘Local’ (to match available emission factors) (Table 5.11).

[8] Emission factors are used to estimate the total weight (grams, kilograms, etc) of a substance emitted by a source. Sometimes, emission factors exist only for a group of substances – for example, total volatile organic compounds (VOCs). Speciation factors provide the percent of the total group emissions that are attributable to specific substances in the group. For example, formaldehyde and acetaldehyde are part of the VOC group.

[9] **Statistics Canada. 2011.** Population ecumene file (http://www.statcan.gc.ca/pub/92-159-g/2011001/use-utiliser-eng.htm)

[10] Statistics Canada 2011. Dissemination Block population file. http://www12.statcan.gc.ca/census-recensement/2011/ref/dict/geo014-eng.cfm

5.1. Vehicle-kilometers travelled by province and vehicle type

	Vehicles up to 4.5 tonnes	Vehicles 4.5 to 14.9 tonnes	Trucks 15 tonnes or more
		millions	
NL	4,367.6	41.1	225.1
PE	1,279.9	11.0	38.8
NS	9,370.2	180.9	500.4
NB	7,765.2	60.3	137.5
PQ	68,133.1	1,031.0	3,563.3
ON	116,076.7	1,719.3	8,006.4
MB	10,027.3	160.1	1,528.9
SK	11,007.3	529.2	1,224.3
AB	41,672.1	2,617.1	5,421.0
BC	33,310.1	1,891.4	585.3
YT	353.8	34.2	122.0
NT	236.6	16.0	61.8
NU	26.3	0.9*	3.07*

*estimated using national average percentage of vehicles in each category (0.03 x 49.1 for vehicles 4.5 to 14.9 tonnes, and 0.1 x 49.1 for vehicles 15 tonnes or more).

Source: Statistics Canada. 2009. Canadian Vehicle Survey: Annual 2009

5.2. Vehicle-kilometers travelled by type of vehicle and road type

	Vehicles up to 4.5 tonnes	Vehicles 4.5 to 14.9 tonnes	Trucks 15 tonnes or more
		Millions VKT *(percent)*	
Roads > 80km speed limit	156,529.9 *(51.7)*	4,274.5 *(51.9)*	15,376.0 *(72.4)*
All other roads	146,429.5 *(48.3)*	3,967.0 *(48.1)*	5,854.9 *(27.6)*
Total - All roads	302,959.4	8,241.5	21,230.9

Roads with 80km speed limit or more = DMTI category 1, 2 and 3

All other roads = DMTI category 4 and 5

Source: Statistics Canada. 2009. Canadian Vehicle Survey: Annual 2009

5.3. Vehicle-kilometers travelled by vehicle type and fuel type

	Vehicles up to 4.5 tonnes	Vehicles 4.5 to 14.9 tonnes	Trucks 15 tonnes or more
		Millions VKT *(percent)*	
Gasoline	292,783.0 *(96.0)*	1,080.7 *(13.0)*	8.1* *(0.03)*
Diesel	10,336.6 *(3.4)*	7,146.0 *(86.2)*	21,400.1 *(99.9)*
Other fuel type	456.5 *(0.5)*	67.9** *(0.8)*	8.1* *(0.03)*
Total - all fuel types	303,576.1	8,294.6	21,416.3

*estimated - difference between total reported and subtotal for diesel, divided by 2 (evenly assigned to gas and other)

**estimated - difference between total reported and subtotal for gasoline and diesel

Source: Statistics Canada. 2009. Canadian Vehicle Survey: Annual 2009

Table 5.4 Emission factors by vehicle, road and fuel type for total volatile organic compounds

Vehicle Type	Road type	Fuel type	Emission type	Emission Factor (g/VKT)
Light (up to 4.5 tonnes)	Local	Gas	Exhaust	1.45
Light (up to 4.5 tonnes)	Local	Diesel	Exhaust	0.513
Light (up to 4.5 tonnes)	Local	Other	Exhaust	1.73
Light (up to 4.5 tonnes)	Highway	Gas	Exhaust	1.24
Light (up to 4.5 tonnes)	Highway	Diesel	Exhaust	0.31
Light (up to 4.5 tonnes)	Highway	Other	Exhaust	1.51
Heavy (over 4.5 tonnes)	Local	Gas	Exhaust	4.77
Heavy (over 4.5 tonnes)	Local	Diesel	Exhaust	1.56
Heavy (over 4.5 tonnes)	Local	Other	Exhaust	5.09
Heavy (over 4.5 tonnes)	Highway	Gas	Exhaust	2.88
Heavy (over 4.5 tonnes)	Highway	Diesel	Exhaust	0.941
Heavy (over 4.5 tonnes)	Highway	Other	Exhaust	3.07
Light (up to 4.5 tonnes)	Local	Gas	Evaporation	0.535
Light (up to 4.5 tonnes)	Local	Diesel	Evaporation	--
Light (up to 4.5 tonnes)	Local	Other	Evaporation	1.07
Light (up to 4.5 tonnes)	Highway	Gas	Evaporation	0.241
Light (up to 4.5 tonnes)	Highway	Diesel	Evaporation	--
Light (up to 4.5 tonnes)	Highway	Other	Evaporation	0.483
Heavy (over 4.5 tonnes)	Local	Gas	Evaporation	2.91
Heavy (over 4.5 tonnes)	Local	Diesel	Evaporation	--
Heavy (over 4.5 tonnes)	Local	Other	Evaporation	5.81
Heavy (over 4.5 tonnes)	Highway	Gas	Evaporation	2.15
Heavy (over 4.5 tonnes)	Highway	Diesel	Evaporation	--
Heavy (over 4.5 tonnes)	Highway	Other	Evaporation	4.29

Source: Environment Australia. 2000. Emissions Estimation Technique Manual for Aggregated Emissions from Motor Vehicles – Version 1.0

Table 5.5 Emission factors by vehicle, road and fuel type for particulate matter (PM_{10})

Vehicle Type	Road type	Fuel type	Emission type	Emission Factor (g/VKT)
Light (up to 4.5 tonnes)	Local	Gas	Exhaust	0.00932
Light (up to 4.5 tonnes)	Local	Diesel	Exhaust	0.148
Light (up to 4.5 tonnes)	Local	Other	Exhaust	0.00329
Light (up to 4.5 tonnes)	Highway	Gas	Exhaust	0.00513
Light (up to 4.5 tonnes)	Highway	Diesel	Exhaust	0.0813
Light (up to 4.5 tonnes)	Highway	Other	Exhaust	0.00181
Heavy (over 4.5 tonnes)	Local	Gas	Exhaust	0.12
Heavy (over 4.5 tonnes)	Local	Diesel	Exhaust	0.584
Heavy (over 4.5 tonnes)	Local	Other	Exhaust	0.0278
Heavy (over 4.5 tonnes)	Highway	Gas	Exhaust	0.066
Heavy (over 4.5 tonnes)	Highway	Diesel	Exhaust	0.321
Heavy (over 4.5 tonnes)	Highway	Other	Exhaust	0.0153

Source: Environment Australia. 2000. Emissions Estimation Technique Manual for Aggregated Emissions from Motor Vehicles – Version 1.0

Table 5.6 Emission factors by vehicle, road and fuel type for lead

Vehicle Type	Road type	Fuel type	Emission type	Emission Factor (g/VKT)
Light (up to 4.5 tonnes)	Local	Unleaded fuel	Exhaust	0.000118
Light (up to 4.5 tonnes)	Highway	Unleaded fuel	Exhaust	0.000082
Heavy (over 4.5 tonnes)	Local	Unleaded fuel	Exhaust	0.00023
Heavy (over 4.5 tonnes)	Highway	Unleaded fuel	Exhaust	0.000161

Source: Environment Australia. 2000. Emissions Estimation Technique Manual for Aggregated Emissions from Motor Vehicles – Version 1.0

Table 5.7 Speciation factors for total volatile organic compounds estimate

Substance	Fuel Type	Emission Type	Speciation Factor (kg/kg)
Acetaldehyde	Gas	Exhaust	0.00437
Acetaldehyde	Gas	Evaporation	--
Acetaldehyde	Diesel	Exhaust	0.155
Acetaldehyde	Other	Exhaust	0.000615
Benzene	Gas	Exhaust	0.0658
Benzene	Gas	Evaporation	0.017
Benzene	Diesel	Exhaust	0.0101
Benzene	Other	Exhaust	0.00000943
1,3-Butadiene	Gas	Exhaust	0.00649
1,3-Butadiene	Gas	Evaporation	0.0018
1,3-Butadiene	Diesel	Exhaust	0.00115
1,3-Butadiene	Other	Exhaust	0.0000552
Ethylbenzene	Gas	Exhaust	0.015
Ethylbenzene	Gas	Evaporation	0.0019
Ethylbenzene	Diesel	Exhaust	--
Ethylbenzene	Other	Exhaust	--
Formaldehyde	Gas	Exhaust	0.0156
Formaldehyde	Gas	Evaporation	--
Formaldehyde	Diesel	Exhaust	0.0826
Formaldehyde	Other	Exhaust	0.00178

Source: Environment Australia. 2000. Emissions Estimation Technique Manual for Aggregated Emissions from Motor Vehicles – Version 1.0

Table 5.8 Speciation factors for particulate matter (PM_{10})

Substance	Fuel Type	Emission Type	Speciation Factor (kg/kg)
Cadmium	Gas	Exhaust	
Cadmium	Diesel	Exhaust	0.0006
Cadmium	Other	Exhaust	
Hexavalent Chromium	Gas	Exhaust	0.00003
Hexavalent Chromium	Diesel	Exhaust	0.00003
Hexavalent Chromium	Other	Exhaust	0.0055
Lead	Diesel	Exhaust	0.0001
Lead	Other	Exhaust	0.0005
Nickel	Gas	Exhaust	0.0001
Nickel	Diesel	Exhaust	
Nickel	Other	Exhaust	0.0055

Source: Environment Australia. 2000. Emissions Estimation Technique Manual for Aggregated Emissions from Motor Vehicles – Version 1.0

Table 5.9 Speciation for diesel fine particulates (diesel $PM_{2.5}$) from total particulate matter (PM_{10})

Substance	Assumed speciation factor	Evidence in literature	Reference
$PM_{2.5}$	0.5 of total PM_{10}	Analysis of $PM_{2.5}/PM_{10}$ ratio for Canadian NAPS (National Air Pollution Surveillance) monitors showed average ratio across all stations to be 0.49.	Brook JR, Dann TF and Burnett RT (1997) Journal of the Air and Waste Management Association, V 47 No 1 pp 2-19.
		Analysis of $PM_{2.5}/PM_{10}$ ratio for monitoring stations in Germany, Sweden and the Netherlands showed ratios ranging from 0.54 to 0.68.	Cyrys J, Heinrich J, Hoek G et al. (2003) Journal of Exposure Analysis and Environmental Epidemiology, V 13 pp 134-143.
Diesel $PM_{2.5}$	0.12 of total $PM_{2.5}$	Diesel exhaust was estimated to contribute 8% to total $PM_{2.5}$ in Toronto.	Brook JR, Poirot RL, Dann TF et al. (2007) Journal of Toxicology and Environmental Health Part A, V 70 pp 191-199.
		Diesel exhaust was estimated to contribute 16% (+/- 7%) to total $PM_{2.5}$ in the southeastern United States.	Zheng M, Cass GR, Schauer JJ et al. (2002) Environmental Science and Technology V 36 No 11 pp 2361-2371.
		Diesel exhaust was estimated to contribute 18% to total $PM_{2.5}$ in Seattle.	Report by Keill and Maykut (2003) cited in Levelton Consultants Ltd (2007) Air Toxics Emission Inventory and Health Risk Assessment – Summary Report for the Greater Vancouver Regional District and Environment Canada.
		Diesel exhaust was estimated to contribute between 10% and 14% to total $PM_{2.5}$ in Seattle.	Wu C, Wu S, Wu Y et al. (2009). Environment International V 35 pp 516-522.

Table 5.10 Percent of provincial and territorial populations inside and outside the national population ecumene

Province/Territory	Population inside ecumene	Population outside ecumene
Alberta	99.2	0.8
British Columbia	98.9	1.1
Manitoba	96.4	3.6
New Brunswick	98.9	1.1
Newfoundland and Labrador	96.9	3.1
Northwest Territories	83.3	16.7
Nova Scotia	99.6	0.4
Nunavut	88.2	11.8
Ontario	99.7	0.3
Prince Edward Island	100.0	0.0
Quebec	99.5	0.5
Saskatchewan	96.3	3.7
Yukon Territory	83.9	16.1

Table 5.11 Provincial road length by aggregated categories 'Highway' and 'Local'

	Length of Roads (km)	
Province	Highway (> 80 km speed limit)	Local
British Columbia	14,226	209,389
Alberta	33,106	222,303
Saskatchewan	32,929	242,166
Manitoba	18,694	92,979
Ontario	21,792	287,763
Quebec	25,915	235,863
New Brunswick	9,913	59,539
Nova Scotia	2,048	50,345
Prince Edward Island	3,583	3,814
Newfoundland/Labrador	7,229	22,572
Yukon	3,499	12,499
Northwest Territories	2,048	12,472
Nunavut	-	1,352

'Highway (>80 km speed limit)' combines DMTI categories Expressway, Primary Highway and Secondary Highway; 'Local" combines DMTI categories Major Road and Local Road

Source: Statistics Canada. 2009. Canadian Vehicle Survey: Annual 2009
